# POSTN Secretion by Extracellular Matrix Cancer-Associated Fibroblasts (eCAFs) Correlates with Poor ICB Response *via* Macrophage Chemotaxis Activation of Akt Signaling Pathway in Gastric Cancer

**DOI:** 10.14336/AD.2023.0503

**Published:** 2023-12-01

**Authors:** Tingting You, Hui Tang, Wenjing Wu, Jingxi Gao, Xuechun Li, Ningning Li, Xiuxiu Xu, Jiazhang Xing, Hui Ge, Yi Xiao, Junchao Guo, Bin Wu, Xiaoyi Li, Liangrui Zhou, Lin Zhao, Chunmei Bai, Qin Han, Zhao Sun, Robert Chunhua Zhao

**Affiliations:** ^1^Department of Oncology, Peking Union Medical College Hospital, Chinese Academy of Medical Sciences and Peking Union Medical College, Beijing, China.; ^2^Institute of Basic Medical Sciences Chinese Academy of Medical Sciences, School of Basic Medicine Peking Union Medical College, Peking Union Medical College Hospital, Center of Excellence in Tissue Engineering Chinese Academy of Medical Sciences, Beijing Key Laboratory, Beijing, China.; ^3^Department of Stomatology Center, Xiangya Hospital, Central South University, Changsha, China.; ^4^Academician Workstation for Oral-Maxillofacial Regenerative Medicine, Central South University, Changsha, China.; ^5^Department of General Surgery, Peking Union Medical College Hospital, Chinese Academy of Medical Sciences and Peking Union Medical College, Beijing, China.; ^6^Department of Pathology, Peking Union Medical College Hospital, Chinese Academy of Medical Sciences and Peking Union Medical College, Beijing, China.; ^7^School of Life Sciences, Shanghai University, Shanghai, China

**Keywords:** CAFs, macrophage chemotaxis, ICB resistance, gastric cancer

## Abstract

Immune checkpoint blockade (ICB) therapy has revolutionized cancer treatment, but its clinical benefit is limited in advanced gastric cancer (GC). Cancer-associated fibroblasts (CAFs) have been reported to be associated with ICB resistance, but the underlying mechanism has not been fully elucidated. Our previous single-cell RNA-seq analysis of GC revealed that POSTN+FAP+ extracellular matrix CAFs (eCAFs) communicate with macrophages. Here, we evaluated the correlation between eCAFs and ICB response in TCGA-STAD and real-world cohorts. Immune infiltration analysis and correlation analysis were performed to assess the relationship between eCAFs and macrophages. We first confirmed a negative correlation between the abundance of eCAFs and the overall response rate (ORR) to anti-PD-1 treatment in TCGA-STAD and real-world GC cohorts. Overexpression of POSTN in CAFs enhanced macrophage chemotaxis, while POSTN interference showed the opposite effect *in vitro* and *in vivo*. Furthermore, the cell density of POSTN+ CAFs was positively correlated with the infiltration level of CD163+ macrophages in GC patient tissues. The results demonstrated that POSTN secreted by CAFs enhances macrophage chemotaxis by activating the Akt signaling pathway in macrophages. Additionally, we found that POSTN+FAP+ eCAFs may exist in multiple solid tumors and are associated with ICB resistance. eCAFs promote macrophage chemotaxis through the secretion of POSTN, thereby leading to ICB resistance. High expression of POSTN is likely to predict a poor response to ICB. POSTN downregulation may be considered as a candidate therapeutic strategy to improve ICB efficacy.

## INTRODUCTION

Gastric cancer (GC) is the third leading cause of cancer-related deaths worldwide [1]. Immune checkpoint blockade (ICB) therapy has shed light on cancer treatment for a variety of solid tumours, but the benefit of anti-PD-1 monotherapy provides a survival benefit in less than 20% of advanced GC patients [2]. Thus, the mechanism underlying ICB resistance remains unclear, and the tumour immune microenvironment [3] has emerged as a key factor [4].

The tumour microenvironment (TME) is an important factor in ICB resistance. Cancer-associated fibroblasts (CAFs) are the most abundant cell type in the TME, and they play a key role in tumorigenesis, metastasis, cancer stem cell renewal, chemoresistance, and immune escape [5, 6]. CAFs affect immune cell infiltration, intratumoral migration, CD8+ T-cell exhaustion, and macrophage polarization [7]. Therefore, CAFs play an important role in the formation of an immunosuppressive TME [7]. Several studies have found that the abundance of CAFs is associated with ICB resistance [8, 9].

CAFs have been proven to be highly heterogeneous, and some CAF subsets are related to ICB resistance. Subgroups associated with ICB resistance may differ between different solid tumour types. In breast cancer, FAP+CAFs are divided into 8 subgroups, among which the TGF-β signalling and extracellular matrix (ECM) -related subgroups are related to primary resistance to immunotherapy [10]. In non-small cell lung cancer (NSCLC), CAF subpopulations expressing Meflin were also relevant to the therapeutic efficacy of ICB monotherapy and prolonged survival [11]. The abundance of CAFs positive for LRRC15^+^ (leucine-rich repeat-containing 15) is correlated with poor outcomes following immunotherapy across six cancer types, such as renal cell carcinoma, head and neck cancer, and NSCLC [12]. Galbo et al. identified six CAF subtypes in a pancancer cohort. Among them, multiple subpopulations were involved in ICB resistance. In metastatic bladder cancer patients with a poor response to treatment, pan-myCAF, pan-dCAF, pan-iCAF and pan-pCAF markers were significantly enriched. Pan-myCAF, pan-dCAF, pan-iCAF, pan-pCAF and pan-iCAFs-2 markers were positively enriched in metastatic melanoma patients, and panddCAF markers were positively enriched in metastatic renal cancer [6]. The CAF subpopulations that lead to ICB resistance have been inconclusive in different solid tumour types.

In a previous single-cell RNA-seq analysis of GC, we classified the fibroblast population into four subgroups, including myofibroblasts, pericytes, inflammatory CAFs (iCAFs), and extracellular matrix-related CAFs (eCAFs) [13]. The eCAFs were characterized by the FAP^+^ PDGFRA^+^POSTN^+^CD34^-^αSMA^-^ expression profile. Gene enrichment analysis showed that eCAFs were associated with the extracellular matrix. Analysis of the interactions among four CAF subpopulations and immune cells showed that eCAFs are most closely related to macrophages. Recent studies have suggested that the abundance of macrophages in the TME is negatively related to the ICB response [14, 15]. Therefore, we hypothesized that the abundance of eCAFs may be related to ICB resistance.

In summary, our study revealed that eCAFs are associated with ICB resistance in GC, likely due to their promotion of macrophage chemotaxis through the secretion of POSTN. This study provides new insights into the role of CAFs in ICB resistance and highlights the potential of targeting eCAFs and POSTN as a strategy to enhance the efficacy of ICB in GC patients.

## MATERIALS AND METHODS

### Patient samples

The clinicopathological characteristics and overall response rate (ORR) of 92 patients in a pancancer cohort (including 22 GC patients, Supplementary Table 2) receiving anti-PD-1 therapy were retrospectively collected from Peking Union Medical College Hospital (PUMCH) from 2018 to 2021 (Supplementary Table 1). The data of these patients were used to analyse the relationship between the ORR for ICB therapy and the eCAF abundance. GC tissues that were used to analyse the relationship between eCAFs and macrophages were collected from 95 other patients who underwent surgery at PUMCH from 2014 to 2019 (Supplementary Table 3). All patients were histologically diagnosed with GC or other cancers by a pathologist from PUMCH. TNM stage was classified according to the criteria of version 8 AJCC. ORR for the whole patient cohort was assessed using RECIST 1.1. Overall survival (OS) was calculated from the date of diagnosis until the date of death from any cause. All patients were followed up by outpatient service or telephone every three months until August 2022. The study was approved by the Ethics Committee of the Chinese Academy of Medical Sciences and Peking Union Medical College. All patients signed written informed consent forms.

### Bioinformatic analysis of the TCGA database

Gene expression data and clinical information from TCGA-stomach adenocarcinoma (STAD) patients (https://portal.gdc.cancer.gov) were downloaded through the TCGA biolinks R package. Pancancer data were downloaded from XENA-TCGA_GTEx (https://xenabrowser.net/datapages/). Transcripts per million reads (TPM) were transformed to log2 (TPM + 1) for further analysis. High and low expression groups were determined according to the median values of POSTN and FAP. The potential response to ICB was predicted by the Tumour Immune Dysfunction and Exclusion (TIDE) algorithm. The Kaplan-Meier test and log-rank test were constructed by the survminer and survival packages, respectively. To further evaluate the impact of the expression levels of POSTN and FAP and the clinicopathological characteristics on OS, univariate and multivariate Cox regression analyses were performed. In addition, immune infiltration levels were quantified by using single sample gene set enrichment analysis (ssGSEA).

### Isolation and culture of human adipose tissue-derived mesenchymal stem cells (MSCs)

MSCs were isolated from human adipose tissue collected during liposuction procedures and cultured as previously published methods [16]. Flow cytometry was employed to identify MSCs using cell surface markers (Supplementary Fig. 3).

### Isolation and culture of human GC-derived CAFs (hCAFs)

hCAFs were isolated from human GC tissue and identified by quantitative reverse transcription-polymerase chain reaction (qRT-PCR) and Western blotting (WB). Both procedures were approved by the Ethics Committee of the Chinese Academy of Medical Sciences and Peking Union Medical College. All donors signed an informed consent document.

### Cell culture

The human gastric tumour cell lines (AGS and GC803) and the human myeloid leukaemia mononuclear cell line (THP-1) were acquired from the Cell Resource Center at the Chinese Academy of Medical Sciences and authenticated by the Cell Resource Center. AGS cells were cultured in Dulbecco’s modified Eagle’s medium (DMEM)/F12, GC803 cells were cultured in DMEM (Gibco, Paisley, UK), and THP-1 cells were cultured in RPMI 1640 medium (Gibco, USA). All media were supplemented with 10% foetal bovine serum (FBS) (Gibco, USA), penicillin (100 U/ml), and streptomycin (100 μg/ml) and incubated at 37°C in a humidified atmosphere with 5% CO2.

### Differentiation of THP-1 cells into macrophages

THP-1 cells were differentiated into macrophages by culturing them with 50-ng/ml phorbol myristate acetate (Sigma; P1585) for 48-h, as previously described [17]. Macrophages were identified by detecting cell surface markers.

### Extraction and identification of exosomes

Exosome extraction was performed as previously described [18]. At 70-80% confluence, AGS and GC803 cells were washed with phosphate-buffered saline (PBS) (Gibco, USA) three times and cultured in serum-free medium for 36 h. To remove dead cells, the supernatants were collected and centrifuged for 30 min at 3,000 rpm at room temperature. Exosomes were centrifuged by using ultrafiltration membranes (molecular weight cut-off, 100 kDa) at 4°C and washed with PBS solution twice to collect transparent and colourless liquid. The resultant liquid was filtered using a 0.2 µm filter unit, aliquoted at 1.5 ml per tube and stored at -80°C immediately until use.

The morphology and structure of the exosomes were observed by transmission electron microscopy (HITACHI, Japan). The particle size and concentration of exosomes were determined by Flow NanoAnalysis (Nanofcm, China). Marker protein expression of exosomes was confirmed by WB.

### Uptake of exosomes by MSCs

AGS and GC803 exosomes were fluorescently labelled with 1 μM Dil (Thermo Fisher, USA) and incubated for 10 min. The labelled exosomes were centrifuged at 700,000 × g at 4°C for 40 min. After discarding the supernatant, the exosomes were added to the MSCs. The MSCs were fixed in 4% paraformaldehyde for 10 min and stained with Hoechst 33342 (Thermo Fisher, USA). The cells were observed under a fluorescence microscope [18, 19].

### RNA extraction and qRT-PCR

Total RNA was extracted using TRIzol (Thermo Fisher Scientific; 15596018) and purified with chloroform. Then, RNA was precipitated with isopropanol, washed with 75% ethanol, and resuspended in 30 μl DEPC water. The RNA concentration was measured by Nanodrop spectrophotometry (Thermo Scientific NanoDrop 2000/2000C). Subsequently, 5000 ng of RNA from each sample was reverse transcribed into cDNA. For qRT-PCR, cDNA templates and primers were mixed with SYBR@Premix Ex TaqTM (TaKaRa, Japan) in 96-well plates. qRT-PCR was performed using QuantStudio Design & Analysis Software. The qRT-PCR amplification conditions were as follows: initial denaturation at 95 °C for 10 min, followed by 40 cycles of 95 °C for 10 s and annealing for 40 s at 60 °C. The data for each sample were semi-quantitatively analysed using the 2-ΔΔCT method. The sequences of all primers are listed in Supplementary Table 4.

### Western blot

WB was performed as previously described[18]. Briefly, the samples were washed twice with precooled PBS and lysed in RIPA buffer (Solarbio, China) with 1-mM phenylmethanesulfonyl fluoride (PMSF) and 1 mM protease inhibitor (Beyotime, China). The protein concentration was determined by BCA assay (Beyotime, China). Equal amounts of proteins were separated on 10% sodium dodecyl sulfate (SDS)-polyacrylamide gel electrophoresis (PAGE) [20] gels and transferred to polyvinylidene fluoride (PVDF) membranes (Millipore, USA). After protein transfer, the membranes were blocked in 3% or 5% milk solution (PBS containing 0.05% Tween-20, PBS-T) for 1-h at room temperature with gentle agitation, incubated in primary antibody solution overnight at 4-°C, and subsequently incubated in secondary antibodies for-1 h at room temperature. Primary antibodies used in WB, including those against GAPDH (CST #5174, Danvers, MA, USA), PDGFRα (CST #3174), MMP2 (CST #40994), HSP90 (CST #4877), HSP70 (CST #4873) and β-Actin (CST; 4970#), were purchased from Cell Signaling Technology (Danvers, Massachusetts, USA). Periostin (Abcam; ab79946) was purchased from Abcam (Cambridge, Massachusetts, USA), and exosome specific-anti-CD63 (Invitrogen; TS63) was purchased from Invitrogen SuperScriptII (Carlsbad, CA, USA). The secondary antibodies used in WB were goat anti-rabbit IgG (H+L) HRP (MultiSciences, China; 70-GAR0072) and goat anti-mouse IgG (H+L) HRP (MultiSciences, China; 70-GAM0072). Finally, chemi-luminescence was detected by chemiluminescence ECL detection reagent (Millipore, USA).

### Lentiviral particle transduction

Lentiviral transduction particles of POSTN-siRNA (GCTTGGGACAACTTGGATTCT) and negative control (si-NC) were chemically synthesized and incorporated into the LV3 (H1/GFP&Puro) vector by GenePharma (Shanghai, China). For stable POSTN overexpression (OE), lentiviral transduction particles of POSTN-OE-RNA (NM_006475.3) and negative control (OE-NC) were also chemically synthesized and incorporated into the LV5 (EF-1a/GFP&Puro). For lentiviral transduction, MSCs were transduced for over 24h using lentiviral supernatants, followed by selection with puromycin. The selection was verified by detection of Green fluorescent protein (GFP) with a fluorescence microscope. Transfection efficiency was also verified by both qRT-PCR and WB.

### Flow cytometry

Flow cytometry was used to identify the immunophenotypes of MSCs as described previously [19]. MSCs were incubated with primary antibodies against CD29, CD31, CD34, CD44, CD73, CD90, and CD106 (BD Biosciences) at 4°C for 1 h. After washing off the primary antibodies, the MSCs were incubated with a fluorescence-labelled secondary antibody (BD Pharmingen, USA) at 4°C for 30 min. The immunopositive cells were quantified using an Accuri C6 Flow Cytometer (BD Biosciences, USA).

### In vitro chemotaxis assay

Chemotaxis of macrophages was analysed via transwell assays as described previously [21]. Macrophages (40,000 cells per transwell in serum-free RPMI 1640 medium) were plated in the upper chamber with 0.1% gelatine-coated membranes, and MSCs or CAFs (100,000 cells per transwell in RPMI 1640 medium with 10% FBS) were plated in the lower chamber. After incubation for 24-hours at 37°C, cells were washed in 1x PBS, fixed in 10% paraformaldehyde for 30 min, and then stained with 0.05% crystal violet for 30 min. Finally, the number of cells that migrated to the bottom of the membrane was assessed by counting in the 4 regions.

### Immunohistochemistry (IHC) and immunofluorescence (IF)

IHC staining was performed using a standard protocol as described previously [19]. The primary antibodies and the dilutions used in IHC staining were anti-Periostin antibody (Abcam, ab79946; 1:75), anti-FAP antibody (Abmart; PA7036S; 1:100), CD163 (Servicebio, GB113152; 1:1000), F4/80 (Servicebio, GB11027; 1:500), PD-1 (Servicebio, GB11338-1; 1:1600), CD68 (Abcam, ab201340; 1:200), and CD8 (Servicebio, GB12068; 1:3000). The secondary antibodies were HRP goat anti-rabbit IgG (Servicebio, GB23303; 1:200), HRP goat anti-mouse IgG (Servicebio, GB23301; 1:200), Alexa Fluor® 488-conjugated goat anti-rabbit IgG (Servicebio, GB25303; 1:400), and Cy3-conjugated goat anti-mouse IgG (Servicebio, GB21301; 1:300). The nuclei were counterstained with DAPI. Staining specificity was determined by applying only the secondary antibody (no primary antibody). IF staining was analysed by HALO image analysis software (Indica Labs). The positive eCAF and macrophage rates were calculated as the percentage of POSTN- or CD163-positive cells divided by the total number of cells in each section, and the positive density was defined as the number of cells positive for POSTN or CD163 per square millimetre (mm^2^).

### Xenograft assay in nude mice

To establish GC subcutaneous xenograft tumour models, female BALB/c nude mice (5-6 weeks old) were subcutaneously injected with 100-μl of cell suspension containing either 5-×-10^6^ GC803 cells mixed with 1-×-10^6^ si-NC-MSCs or 5-×-10^6^ GC803 cells mixed with 1-×-106 si-POSTN-MSCs. Tumour volume was calculated using the formula (width)^2^ ×length/2. The mice were euthanized 30 days after cell inoculation or when the longest dimension of the tumour volume reached 1,500 mm^3^ whichever occurred first. Tumour tissues were formalin fixed and paraffin embedded for histological and IHC examinations as mentioned above. All animal experiments were performed according to protocols approved by the Institutional Animal Care and Use Committee (IACUC) of the Chinese Academy of Medical Sciences.

### Statistical analysis

For continuous variables, the statistical significance of normally distributed variables was estimated by independent Student’s t test, and the statistical significance of nonnormally distributed variables was estimated by the Wilcoxon rank-sum test. For categorical variables, the chi-square test was performed. Spearman’s rank or Pearson’s correlation coefficient was used to determine the relationship between variables. The Kaplan-Meier method was used to assess survival, and the log-rank test was used to make comparisons between two groups. The hazard ratio (HR) was estimated using the Cox proportional hazard regression model. All statistical analyses were carried out via R (v.4.0.2) software packages, and a *p*-value less than 0.05 was considered to indicate statistical significance. All figures were generated using the ggplot2 R package.

**Table 1:** Univariable and multivariable Cox analyses of OS in TCGA-STAD.

Characteristics	Total(N)	Univariate analysis		Multivariate analysis	
		HR (95% CI)	*p*-value	HR (95% CI)	*p*-value
T stage	362				
T1_2	96	Reference			
T3_4	266	1.719 (1.131-2.612)	0.011	1.273 (0.800-2.028)	0.309
N stage	352				
N0	107	Reference			
N+	245	1.925 (1.264-2.931)	0.002	1.896 (1.197-3.004)	0.006
M stage	352				
M0	327	Reference			
M1	25	2.254 (1.295-3.924)	0.004	2.504 (1.382-4.537)	0.002
Age	367				
<=65	163	Reference			
>65	204	1.620 (1.154-2.276)	0.005	1.818 (1.263-2.616)	0.001
Gender	370				
Female	133	Reference			
Male	237	1.267 (0.891-1.804)	0.188		
POSTN	370				
Low	186	Reference			
High	184	1.538 (1.105-2.140)	0.011	1.699 (1.050-2.749)	0.031
FAP	370				
Low	185	Reference			
High	185	1.434 (1.030-1.997)	0.033	0.977 (0.605-1.579)	0.926

## RESULTS

### eCAFs correlate with poor ICB response in GC

**Figure 1.**
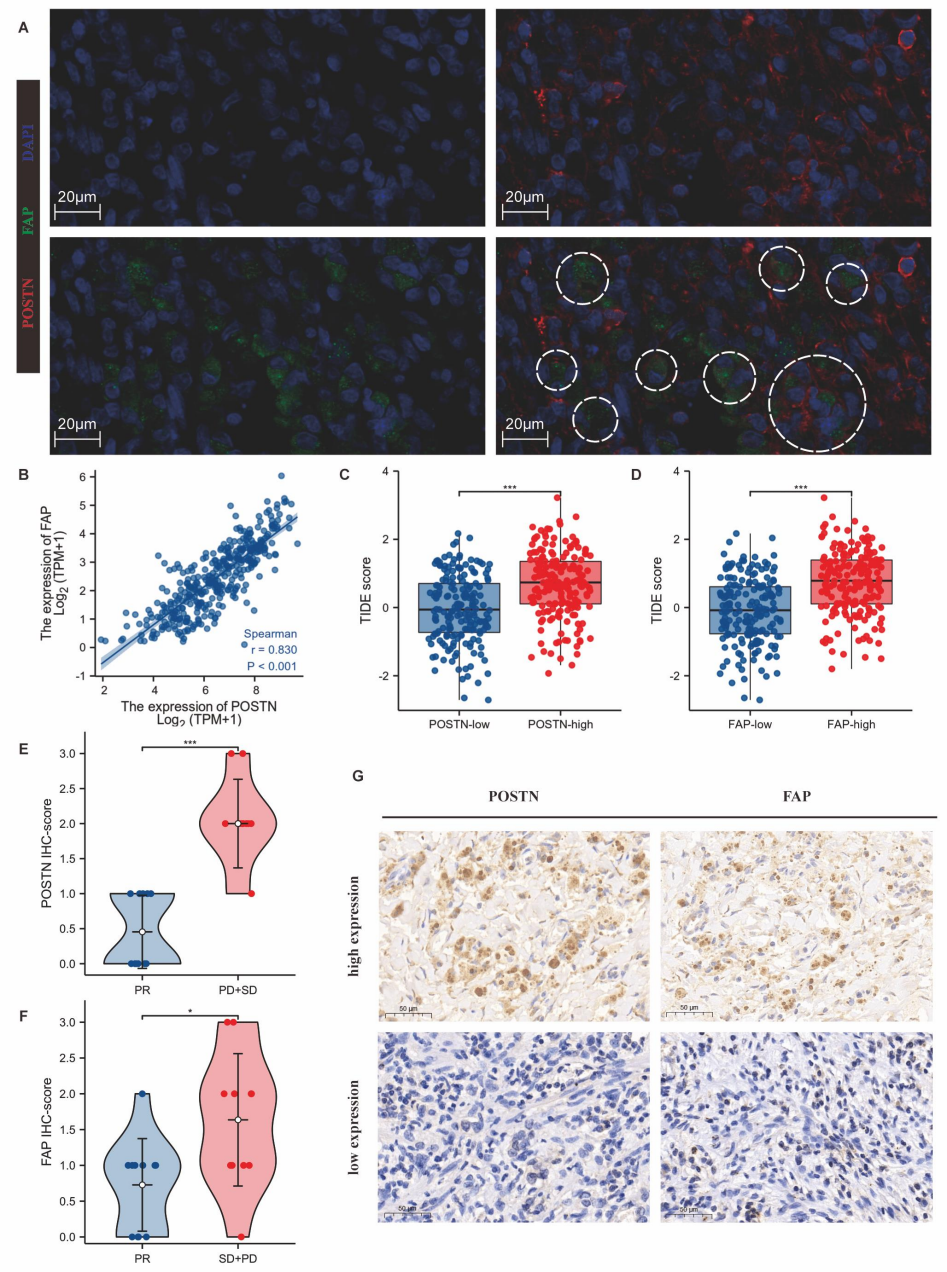
POSTN+FAP+ eCAFs conferred resistance to immune checkpoint blockade (ICB) in GC. (A) IF detection (n = 6) of POSTN and FAP double-positive CAF subpopulations in GC. Red: POSTN; Green: FAP; Blue: nucleus; White circle: typical FAP and POSTN double-positive CAFs. Scale bar: 20 μm. (B) The expression of POSTN was positively correlated with FAP expression in TCGA-STAD (*p*<0.001; Spearman’s rank). (C-D) High expression of POSTN (C) and FAP (D) was correlated with high TIDE scores. Red bars represent higher expression levels, and blue bars represent lower expression levels. ***, *p*<0.001; T test. (E-F) High expression of POSTN (E) and FAP (F) in GC was related to poor response to immunotherapy. PR: partial response; SD: stable disease; PD: progressive disease. Red bars represent the PD and SD groups, and blue bars represent the PR group (***, *p*<0.001; *, *p*<0.05; Wilcoxon rank sum test). (G) IHC staining (n = 22) of POSTN and FAP in GC tissue specimens (×40). Scale bar: 50 µm.

We first confirmed the existence of POSTN^+^FAP^+^ eCAF subpopulations in tumour tissues of GC patients and in the TCGA database. POSTN and FAP were mainly expressed in GC stromal cells (Supplementary Fig.1A-E). In GC tumour tissue sections, there were fibroblast-like cells with double-positive for POSTN and FAP staining according to IF (Fig. 1A). The expression levels of POSTN and FAP showed a significant positive correlation (Fig 1B). Furthermore, GC patients with high expression of POSTN and FAP had shorter OS in TCGA-STAD cohort (Supplementary Fig. 2, Table 1).

**Figure 2.**
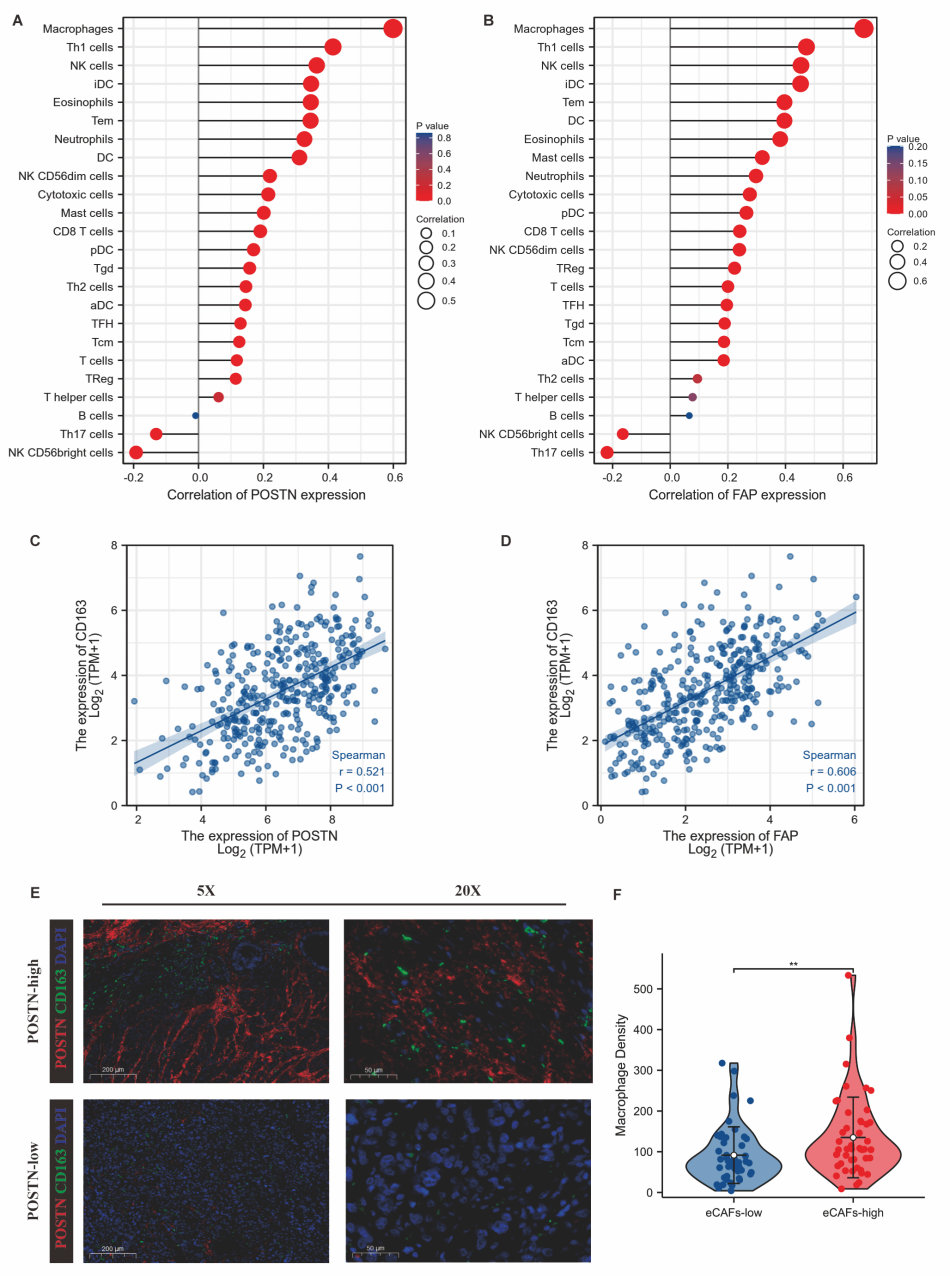
Relationship between eCAFs and macrophage infiltration in GC. (A) Relationship between POSTN (A) and FAP (B) expression and infiltration of immune cells in TCGA-STAD (Spearman’s rank). (C-D) Expression levels of POSTN (C) and FAP (D) were positively correlated with CD163 expression levels in TCGA-STAD (*p*<0.001; Spearman’s rank). (E, F) The density of POSTN-positive cells was positively correlated with that of CD163-positive cells. E: Representative IF (n = 95) was used to detect the density of POSTN-positive and CD163-positive cells; F: statistical data. Red: POSTN; Green: CD163; Blue: DAPI; **, *p*<0.01; Wilcoxon rank sum test.

**Figure 3.**
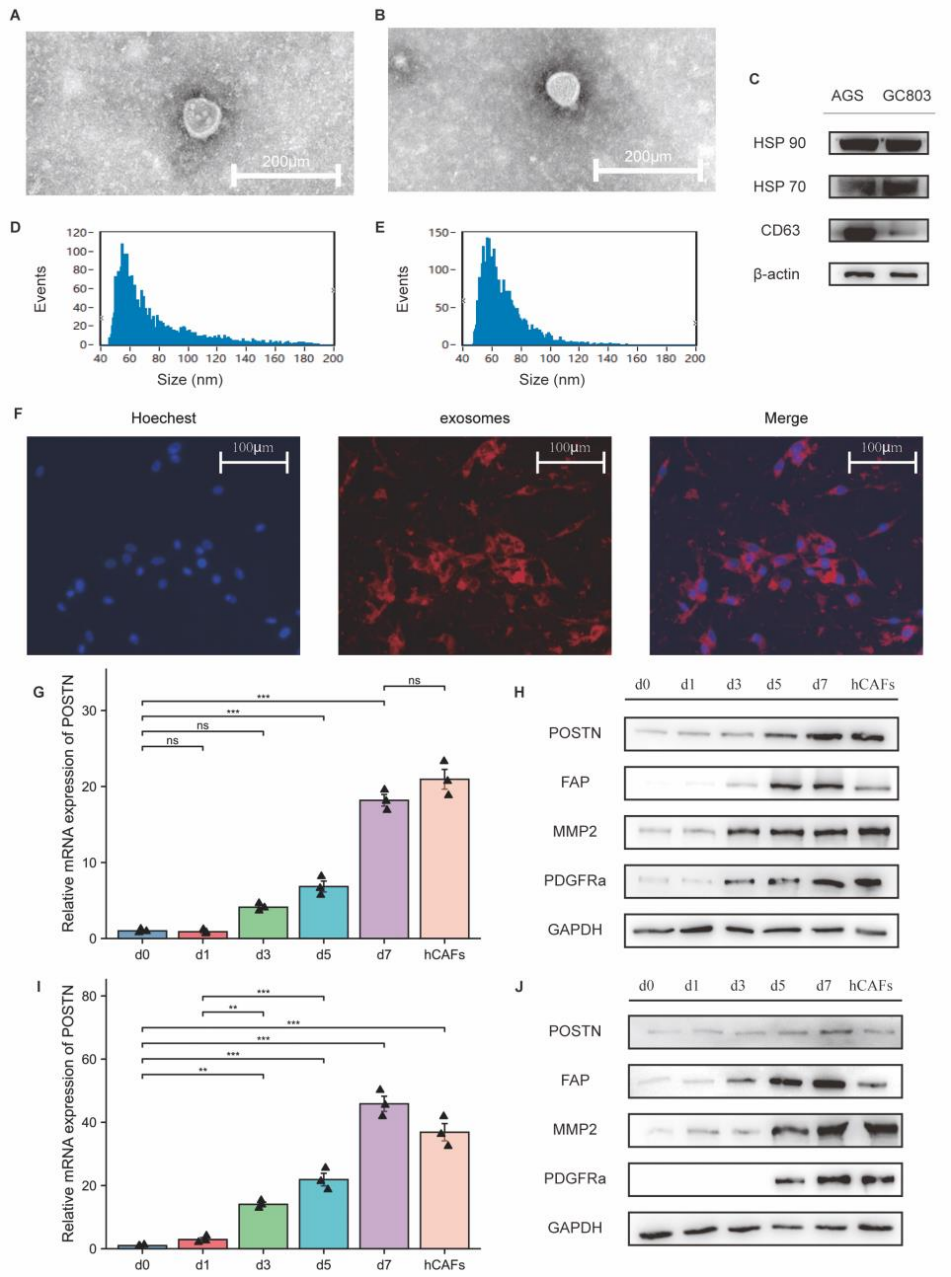
The expression levels of POSTN and FAP in CAFs were upregulated after induction with GC exosomes. (A) The morphology of AGS (A) and GC803 (B) exosomes was detected by transmission electron microscopy. (C) Specific biomarker expression of gastric tumour cell line (AGS and GC803) exosomes detected by WB. (D-E) The diameter of AGS (C) and GC803 (D) exosomes was measured by a nano-flow analyser. (F) Fluorescence microscopy showed that MSCs could take up exosomes (red) into the cytoplasm. Scale bar: 100 μm. (G, I) Quantitative PCR analysis indicated that the relative mRNA expression level of POSTN was significantly upregulated after stimulation by AGS (G) and GC803 exosomes (I). ***, *p*< 0.001; **, *p*-value<0.01; ns, no significant difference (One-way ANOVA). (H, J) Western blot analysis indicated that the protein levels of POSTN, FAP, MMP2, and PDFGRα were significantly upregulated after stimulation with AGS exosomes (H) and GC803 exosomes (J).

In previous studies, we found that POSTN^+^FAP^+^ eCAFs highly expressed genes involved in ECM remodelling, including genes encoding collagens and collagen metabolic enzymes [13]. Moreover, it was reported that ECM-related CAF subsets indicated primary resistance to immunotherapies in breast cancer [22]. Therefore, we first examined the relationship between POSTN level, FAP level, and TIDE score in the TCGA-STAD dataset. High expression of POSTN or FAP was positively correlated with a high TIDE score, indicating a poor response to ICB (Fig. 1C-D). Subsequently, we retrospectively analysed the ORR of 22 GC patients who received immunotherapy at PUMCH (Supplementary Table 2). The high expression of POSTN (*p*<0.001) or FAP (*p*<0.05) was positively correlated with a poor response to treatment (Fig. 1E-G). These results have confirmed that POSTN^+^FAP^+^ eCAFs are distributed in the stromal area and correlate with poor ICB response in GC patients.

### The density of eCAFs may be related to macrophage infiltration in GC.

In our previous work, we identified intercellular communication between eCAFs and macrophages [13]. Macrophage infiltration is one of the critical determinants of ICB resistance [23-25]. We therefore hypothesized that eCAFs promoted resistance to ICB through chemotactic macrophages. The ssGSEA algorithm (Fig. 2A-B) showed that the expression of POSTN and FAP was significantly associated with the immune infiltration of macrophages in TCGA-STAD. The expression of POSTN (R=0.521) and FAP (R=0.606) was correlated with that of CD163 (*p*<0.001) (Fig. 2C-D), which is a characteristic gene of tumour-associated macrophages (TAMs). In 95 surgical specimens of GC patients (baseline characteristics in Supplementary Table 3 and Cox analyses in Table 2) from PUMCH, the positive density of POSTN (the characteristic marker of eCAFs) was positively correlated with the positive density of CD163 (the characteristic marker of TAMs) (Fig. 2E-F). There was no significant correlation between the expression of CD163 and clinical characteristics in GC patients (Supplementary Fig. 4).

**Table 2:** Univariable and multivariable Cox analyses of OS in 95 GC patients.

Characteristics	Total (N)	Univariate analysis		Multivariate analysis	
		HR (95% CI)	*p*-value	HR (95% CI)	*p*-value
Age	95	0.975 (0.923-1.031)	0.376		
T	95		0.998		
T3_4	70	Reference			
T1_2	25	0.000 (0.000-Inf)	0.998		
N	95		0.282		
N+	69	Reference			
N0	26	0.320 (0.040-2.547)	0.282		
M	95		0.167		
M1	22	Reference			
M0	70	0.292 (0.081-1.046)	0.059		
Mx	3	0.000 (0.000-Inf)	0.999		
stage	95		0.473		
IV	33	Reference			
III	46	0.456 (0.130-1.604)	0.221		
I_II	16	0.000 (0.000-Inf)	0.998		
Lauren type	92		0.434		
diffuse type	37	Reference			
intesnial type	32	0.417 (0.084-2.075)	0.285		
mixed type	23	0.370 (0.044-3.087)	0.358		
Her-2 IHC staining	90		1.000		
negative	75	Reference			
median	11	0.982 (0.124-7.792)	0.986		
positive	4	0.000 (0.000-Inf)	0.998		
CD163 Positive Cell Density	95	1.005 (1.001-1.010)	0.020	1.005 (1.001-1.009)	0.020
postn Positive Cell Density	95	1.001 (1.000-1.002)	0.037	1.001 (1.000-1.002)	0.035

### Isolation of GC-derived hCAFs and induction of MSC-derived CAFs in vitro.

We isolated hCAFs from human GC specimens. Because of the limitations regarding hCAF proliferative capacity and GC tissue volume, we established a method to induce MSCs to differentiate into CAFs by utilizing gastric tumour cell exosomes in vitro [18, 21]. However, whether CAFs induced in vitro express characteristic genes of eCAFs remains to be determined. The characteristics of gastric tumour cell exosomes and MSCs were confirmed and were similar to those previously described (Fig. 2A-E; Supplementary Fig. 3) [18, 21]. After adding gastric tumour cell exosomes to the culture medium, exosomes were taken up by MSCs (Fig. 2F). After culture for 7 days, qRT-PCR and WB assays showed that the expression of POSTN, FAP, MMP2 and PDGFα in the gastric tumour cell exosome-treated group was gradually increased compared with that in the MSC control group (Fig. 2G-J). Moreover, the expression levels of the above genes in exosome-induced CAFs were similar to those in hCAFs (Fig 2. G-J). Therefore, we used exosome-induced CAFs in the following experiments.

**Figure 4.**
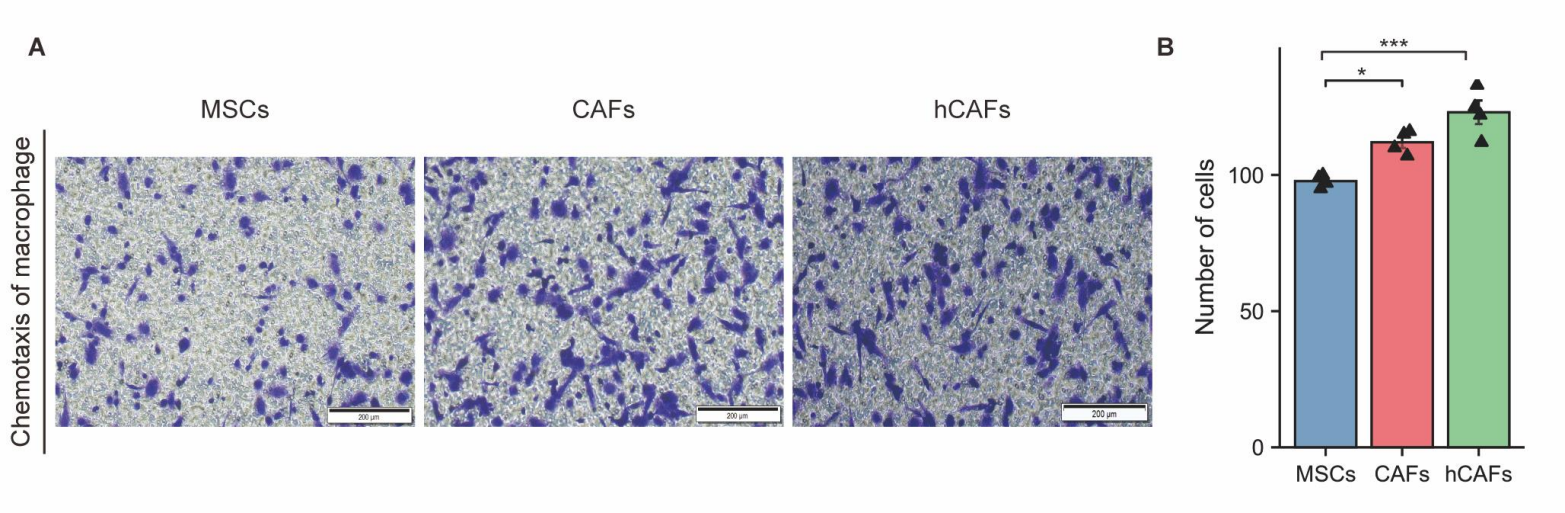
The ability of CAFs to induce macrophage chemotaxis was stronger than that of MSCs. (A) Transwell assay of chemotactic macrophages (n = 4); (B) Statistical data. ***, *p*< 0.001; *, *p*< 0.05; ns, no significant difference; One-way ANOVA.

### POSTN plays a key role in eCAFs-mediated induction of macrophage chemotaxis.

We compared the chemotactic ability of MSCs, CAFs induced by exosomes, and hCAFs isolated from GC specimens to recruit macrophages in vitro. CAFs induced by exosomes (*p*<0.05) and hCAFs (*p*<0.001) recruited significantly more than MSCs (Fig. 4). In TCGA-STAD, patients with high CD163 expression had higher TIDE scores (*p*<0.05) (Supplementary Fig. 5A). Among 22 GC patients who received immunotherapy at PUMCH, patients with high expression of CD68 and CD163 in tumour tissues had a lower ORR (Supplementary Fig. 5B-D, G). However, the positive staining rates of CD8 and PD-1 were not related to ORR (Supplementary Fig. 5E-G). Therefore, a high density of eCAFs and TAMs was related to ICB resistance.

**Figure 5.**
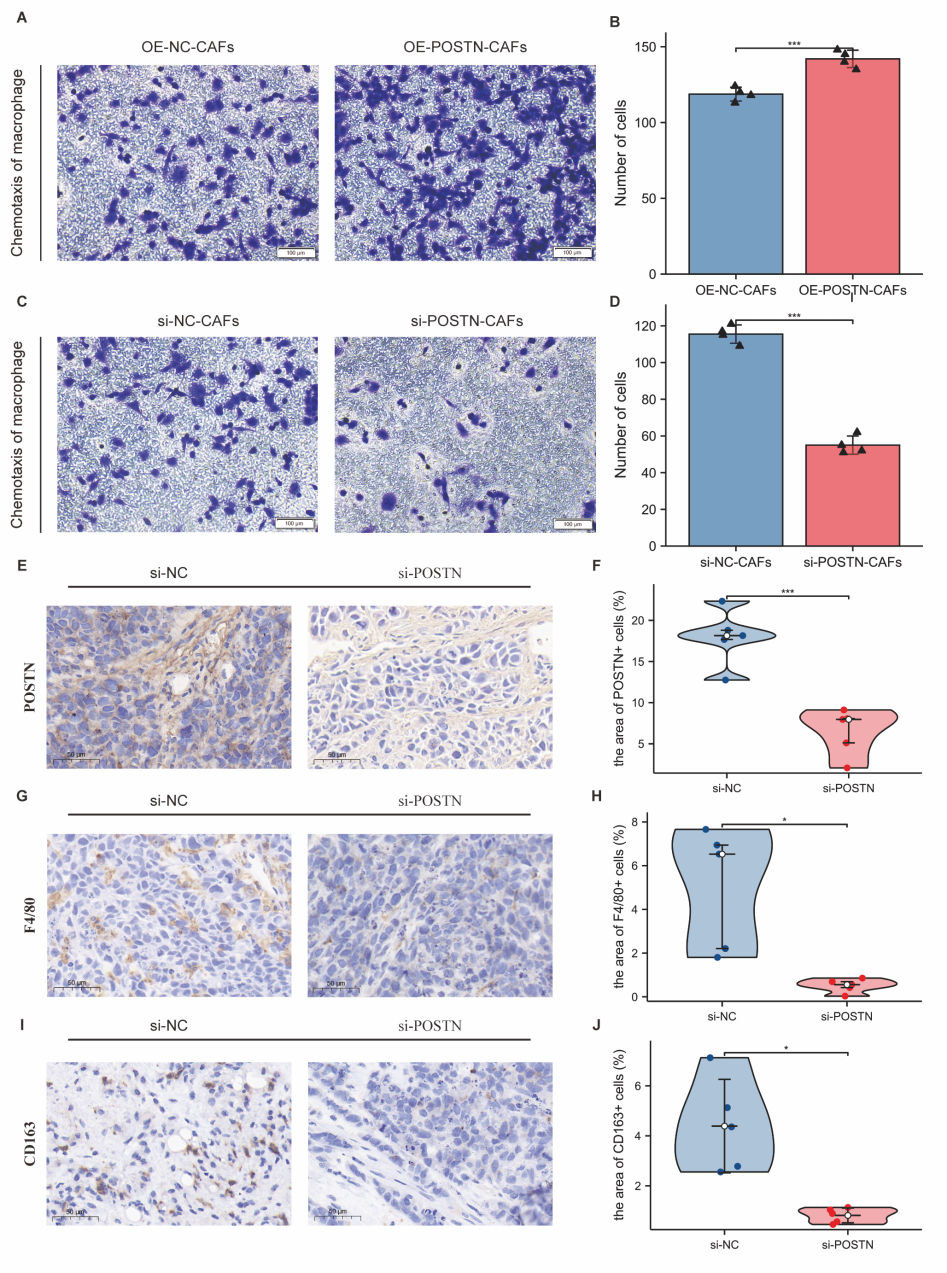
POSTN played a key role in the ability of CAFs to induce macrophage chemotaxis. (A, B) The ability of CAFs overexpressing POSTN (OE-POSTN-CAFs) to induce macrophage chemotaxis was stronger than that of the negative control group (OE-NC-CAFs). (A) Transwell assay of chemotactic macrophages (n = 4)(CAFs induced by exosomes from AGS cell line). (B) Statistical data. ***, *p*< 0.001; T test. (C-D) The ability of CAFs with POSTN knockdown (si-POSTN-CAFs) to induce macrophage chemotaxis was stronger than that of the control group (si-NC-CAFs). (C) Transwell assay of chemotactic macrophages (n = 4)(CAFs induced by exosomes from AGS cell line). (D) Statistical data. ***, *p*< 0.001; T test. (E-F) The effect of si-POSTN-CAFs on the expression of POSTN in the GC803 subcutaneous xenograft tumour model. E: Immunohistochemical staining of POSTN (n = 5). (F) Statistical data. **, *p*< 0.01; Wilcoxon rank sum test. (G-H) The effect of si-POSTN-CAFs on the expression of F4/80 in the GC803 subcutaneous xenograft tumour model. (E) Immunohistochemical staining of F4/80 (n = 5); (F) Statistical data. *, *p*< 0.05; Wilcoxon rank sum test. (I-J) The effect of si-POSTN-CAFs on the expression of CD163 in the GC803 subcutaneous xenograft tumour model. (E) Immunohistochemical staining of CD163 (n = 5). (F) Statistical data (*, *p*< 0.05)(Wilcoxon rank sum test).

POSTN is the characteristic gene of eCAFs. Since the density of POSTN+ cells was positively correlated with that of CD163+ macrophages (Fig. 2E-F), we speculated that POSTN might be the functional molecule in eCAFs that regulates chemotactic macrophages. POSTN-overexpressing CAFs (OE-POSTN-CAFs), POSTN-silenced CAFs (si-POSTN-CAFs) and negative controls were derived from MSCs transfected with lentivirus vectors carrying different sequences and then induced by GC exosomes (Supplementary Fig. 7A-D). The transwell assay showed that the ability of OE-POSTN-CAFs to recruit macrophages was significantly enhanced compared with that to OE-NC-CAFs (*p*<0.001) (Fig. 5A, Supplementary Fig. 8A). Moreover, the ability of si-POSTN-CAFs to recruit macrophages was significantly decreased compared with that to si-NC-CAFs (*p*<0.001) (Fig. 5B, Supplementary Fig. 8B). To further observe the effect of POSTN in a GC mouse xenograft model in vivo, GC803 cells mixed with si-NC-MSCs or si-POSTN-MSCs were subcutaneously injected into nude mice. After 6 weeks, subcutaneous tumours were harvested for IHC of POSTN, F4/80, and CD163. The positive areas of F4/80 and CD163 were significantly smaller in the si-POSTN group than in the control group (*p*<0.05) (Fig. 5E-J). These results demonstrated that POSTN secreted by eCAFs plays a key role in macrophage chemotaxis.

### POSTN significantly promoted macrophage chemotaxis by activating the Akt signalling pathway in macrophages.

**Figure 6.**
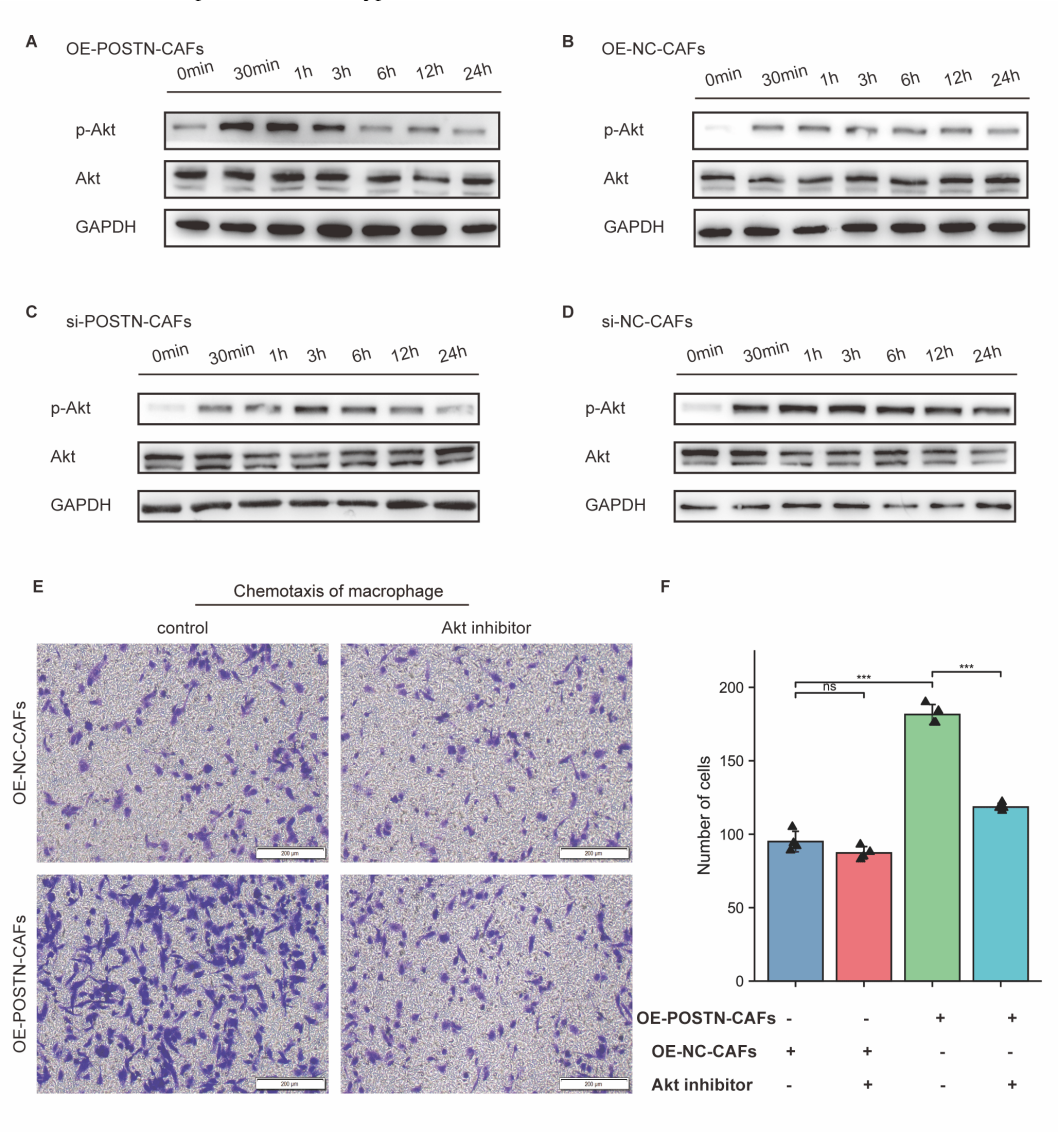
eCAFs significantly promoted macrophage chemotaxis by activating the Akt signalling pathway in macrophages. (A) The effect of POSTN overexpression in CAFs on Akt phosphorylation in macrophages was detected by Western blotting. A: OE-POSTN-CAFs: macrophages treated with conditioned medium from CAFs overexpressing POSTN; B: OE-NC-CAFs: macrophages treated with conditioned medium from control CAFs. (C-D) The effect of POSTN-silenced CAFs on Akt phosphorylation in macrophages was detected by Western blotting. C: si-POSTN-CAFs: macrophages treated with POSTN-silenced CAF-conditioned medium; D: si-NC-CAFs: macrophages treated with control CAF-conditioned medium. (E) Effect of Akt phosphorylation inhibitor on chemotactic macrophages of CAFs (n = 4). Left top panel: Treatment of macrophages with control CAF conditioned medium with Akt phosphorylation inhibitor or DMSO; left bottom panel: Treatment of macrophages with overexpressing POSTN CAF conditioned medium with Akt phosphorylation inhibitor or DMSO; F: Statistical data. ***, *p*<0.001; ns, no significant difference; T test).

Previous studies have suggested that POSTN secreted by glioblastoma stem cells activates the Akt signalling pathway in macrophages through integrin αvβ3 signalling, leading to macrophage chemotaxis [26]. The level of POSTN was significantly upregulated in OE-POSTN-CAFs conditioned medium compared to control medium (Supplementary Fig. 9). Macrophages were cultured in OE-POSTN-CAF- or OE-NC-CAF-conditioned medium. Akt phosphorylation in macrophages was significantly upregulated in the OE-POSTN-CAF-conditioned medium treatment group, as determined by WB assay (Fig. 6A-B). When compared to that in the si-NC-CAF group, Akt phosphorylation in macrophages was significantly decreased in the si-POSTN-CAF group (Fig. 6C-D), indicating that POSTN could activate the Akt signalling pathway in macrophages. When an Akt phosphorylation inhibitor was added to CAF culture medium, the ability of the cells to recruit macrophages was significantly inhibited (*p*<0.001) (Fig. 6E). The same phenotype was observed when αvβ3 neutralizing antibody was added (Supplementary Fig. 11). Thus, it was confirmed that the chemotactic capacity of POSTN derived from CAFs was also dependent on the Akt signalling pathway.

### POSTN^+^FAP^+^eCAFs are related to ICB resistance across cancers.

Single-cell RNA-seq revealed that the characteristics of some CAF subpopulations were similar in different solid tumours [10]. Therefore, we wondered whether the eCAF subpopulations might be widely present across cancers; we used data from the TCGA database and performed pancancer analysis. The results showed that the expression levels of POSTN and FAP in breast cancer (BRCA), lung adenocarcinoma (LUAD), lung squamous cell carcinoma (LUSC), and digestive system tumours such as colon adenocarcinoma (COAD) and oesophageal cancer (ESCA) were significantly higher than those in normal tissues (*p*<0.001) (Supplementary Fig. 12A-B). A significant positive correlation was also found between the expression of POSTN and FAP in multiple solid tumours (*p*<0.001) (Supplementary Fig. 12C-H). Similar to the results for GC, high expression of POSTN or FAP was significantly positively correlated with a high TIDE score in other solid tumours (Supplementary Fig. 13A-F). Therefore, we speculate that eCAF subpopulations might exist in multiple solid tumours. We retrospectively analysed the ORR of 92 patients who received immunotherapy at PUMCH, including 22 GC patients (Supplementary Table 1). High expression of POSTN (*p*<0.001), FAP (*p*<0.001) and CD163 (*p*<0.001) was positively correlated with a poor response to immunotherapy (Supplementary Fig. 14A-E). These results suggest that the correlation between POSTN^+^FAP^+^eCAFs and ICB resistance may be a universal phenomenon across cancers, providing an important reference for investigators in the future.

## DISCUSSION

The pro-tumour function of CAFs during cancer development makes them promise therapeutic targets for cancer treatment. Due to the heterogeneity of CAFs and the lack of specific surface markers, there are still significant challenges in the application of CAF-targeted therapies [5]. Advances in single-cell RNA-seq technology have made it possible to accurately analyse the characteristics and functions of CAF subsets. Several investigators have reported that there are differences in CAF subgroup-associated ICB efficacy differences in diverse solid tumours. Possible reasons for this discrepancy include the differences in the tumour microenvironment (TME) across solid tumours and the variability in the number of samples across single-cell sequencing studies. Despite these differences, there are also conspicuous similarities. Many studies have reported that the abundance of ECM-related CAFs is related to ICB resistance. We previously classified CAFs into four subgroups in GC [13]. These characteristic genes of eCAFs were similar to other reported CAF subsets related to ICB resistance [10, 12]. Among them, POSTN^+^FAP^+^eCAFs were found to correlate with ICB resistance in both TCGA-STAD and real-world GC cohorts.

According to functional enrichment analysis of differentially expressed genes, eCAFs were mainly enriched in genes related to the regulation of collagen metabolism and extracellular matrix assembly. However, the specific mechanisms by which ECM-related CAFs lead to ICB resistance remain to be fully elucidated. In breast cancer, ECM-related CAFs increase the protein expression of PD-1 and CTLA4 on the surface of CD4+CD25+ T lymphocytes and participate in immunotherapy resistance [10]. In NSCLC, FAP^+^aSMA^+^CAFs produce collagen, form multiple layers in tumours, and prevent T-cell contact with tumour cells [27]. In multiple solid tumours, a high level of LRRC15^+^ CAFs is correlated with poor outcomes following immunotherapy [12]. In summary, ECM-related CAFs may orchestrate immune cell distribution and function in the TME, leading to ICB resistance.

High expression of the secretory protein POSTN is one of the main characteristics of eCAFs. POSTN is a 93 kDa nonstructural extracellular matrix protein that participates in various tumour biological processes, such as proliferation, invasion, matrix remodelling, and the formation of the cancer stem cell niche and premetastatic niche [28, 29]. Zhou et al. reported that POSTN secreted by glioblastoma stem cells recruited M2 tumour-associated macrophages [26]. Moreover, the relationship of POSTN with metastasis and treatment resistance has been confirmed in multiple solid tumour types [30-35].

In our previous studies, we found that eCAFs were closely related to macrophages through gene expression signatures [13]. The close relationship between POSTN and macrophages has been reported in a variety of solid tumours [36-39]. In this study, the positive correlation between eCAFs and TAMs was validated in the TCGA-STAD cohort and 95 GC patients from PUMCH, consistent with the results reported previously. Then, we compared the degree of macrophage chemotaxis to OE-POSTN-CAFs and si-POSTN-CAFs in vitro and found that POSTN played a key role in eCAFs-mediated induction of macrophage chemotaxis. The subcutaneous model of GC in nude mice allowed us to reach the same conclusion. In summary, POSTN+FAP+eCAFs might promote macrophage chemotaxis by secreting POSTN.

Multiple studies have reported that the infiltration of TAMs is associated with resistance to anti-PD1/PD-L1 antibodies and immune escape [23-25, 40]. The mechanisms of TAM-related ICB resistance are under intense investigation. Some well-studied mechanisms are described as follows: (a) TAMs regulate T-cell function via PD-L1 and inhibitory cytokines (such as IL-10 and TGF-β) [41-43]; (b) TAMs mediate the recruitment of immunosuppressive populations (such as Treg cells) and stimulatory populations (such as dendritic cells)[41, 44]; and (c) TAMs regulate the vascular structure and extracellular matrix to exclude T cells from the intratumoural area [15]. Therefore, we speculate that eCAFs might promote ICB resistance through POSTN-mediated macrophage chemotaxis to gastric tumour tissues.

Previous studies have shown that POSTN secreted by glioblastoma stem cells activates the Akt signalling pathway through integrin αvβ3 signalling, leading to macrophage recruitment [26]. We detected changes in the Akt signalling pathway in macrophages treated with OE-POSTN-CAF- or si-POSTN-CAF-conditioned medium in vitro. The results showed that OE-POSTN-CAFs could significantly promote Akt phosphorylation in macrophages. The Akt/PKB family includes Akt 1, 2 and 3. According to previous studies, Akt phosphorylations plays a regulatory role in cell growth, proliferation, survival and metabolism [45, 46]. Studies on fibroblasts and epithelial cells have shown that the Akt isozyme regulates cell migration by regulating the transcription of genes related to migration [47]. To explore the role of Akt2 in macrophage chemotaxis, researchers used small interfering RNA to specifically downregulate the expression of Akt2 in THP-1 cells and mouse peritoneal macrophages. The results showed that the downregulation of Akt2 could suppress both CSF-1 expression and MCP-1 expression, thus significantly weakening macrophage chemotaxis [45]. Therefore, eCAFs might promote macrophage chemotaxis by activating Akt phosphorylation.

Another characteristic marker of eCAFs is FAP. In NSCLC, FAP^+^aSMA^+^CAFs, which prevent T-cell contact with tumour cells, are also characterized by high expression levels of POSTN [27]. Therefore, we speculate that POSTN^+^FAP^+^eCAFs exist in multiple solid tumour types. In the pancancer analysis of the TCGA database, the high POSTN or FAP expression group showed a high TIDE score. For 92 patients in a pancancer cohort receiving immunotherapy in PUMCH, high expression of POSTN and FAP was positively correlated with a low ORR.

CAFs are currently considered to be potential targets for anti-tumor therapy, and their primary mechanism involves the targeting of key signalling pathways, proteins and the extracellular matrix of CAFs [5]. To enhance the efficacy of chemotherapy and immunotherapy, targeted FAP^+^CAFs antibody drug conjugates and CAR-T are currently being studied in animal models and clinical trials [48, 49]. However, the clinical benefit for patients is not well defined. This study revealed the predictive ability of eCAFs to predict the efficacy of immunotherapy in GC and other cancers and provided potential targets for improving the efficacy of immunotherapy. However, there are also some limitations to this study. On the one hand, this is a retrospective analysis based on a small cohort of immunotherapy patients, and the results may be biased. Therefore, large-scale prospective trials are needed to provide clearer evidence. On the other hand, the feasibility of clinical transformation of strategies affecting the interaction between eCAFs and macrophages needs to be further explored.

### Conclusion

The abundance of POSTN^+^FAP^+^eCAFs might play a role in predicting ICB efficacy. POSTN^+^FAP^+^eCAFs promote macrophage chemotaxis by secreting POSTN, leading to ICB resistance. POSTN^+^FAP^+^eCAFs are promising targets for ICB combination therapy.

## Supplementary Materials

The Supplementary data can be found online at: www.aginganddisease.org/EN/10.14336/AD.2023.0503.
