# Supplementary material for: POSTN Secretion by Extracellular Matrix Cancer-Associated Fibroblasts (eCAFs) Correlates with Poor ICB Response via Macrophage Chemotaxis Activation of Akt Signaling Pathway in Gastric Cancer
Source: Aging Dis. 2023 Dec 1;14(6):2177–92. doi: 10.14336/AD.2023.0503 (PMC10676785; doi:10.14336/AD.2023.0503)
Supplement: Supplementary file 1 — The Supplementary data can be found online at: www.aginganddisease.org/EN/10.14336/AD.2023.0503. [file AD-14-6-2177-s.pdf]

SUPPLEMENTARY DATA

POSTN Secretion by Extracellular Matrix Cancer-Associated Fibroblasts (eCAFs) Correlates with Poor ICB Response *via* Macrophage Chemotaxis Activation of Akt Signaling Pathway in Gastric Cancer

Tingting You[1], Hui Tang[1], Wenjing Wu[2], Jingxi Gao[2], Xuechun Li[3,4], Ningning Li[1], Xiuxiu Xu[1], Jiazhang Xing[1], Hui Ge[1], Yi Xiao[5], Junchao Guo[5], Bin Wu[5], Xiaoyi Li[5], Liangrui Zhou[6], Lin Zhao[1*], Chunmei Bai[1*], Qin Han[2*], Zhao Sun[1*], Robert Chunhua Zhao[2,7*]

© 2023. You T et al. Published online at http://www.aginganddisease.org/EN/10.14336/AD.2023.0503

SUPPLEMENTARY DATA

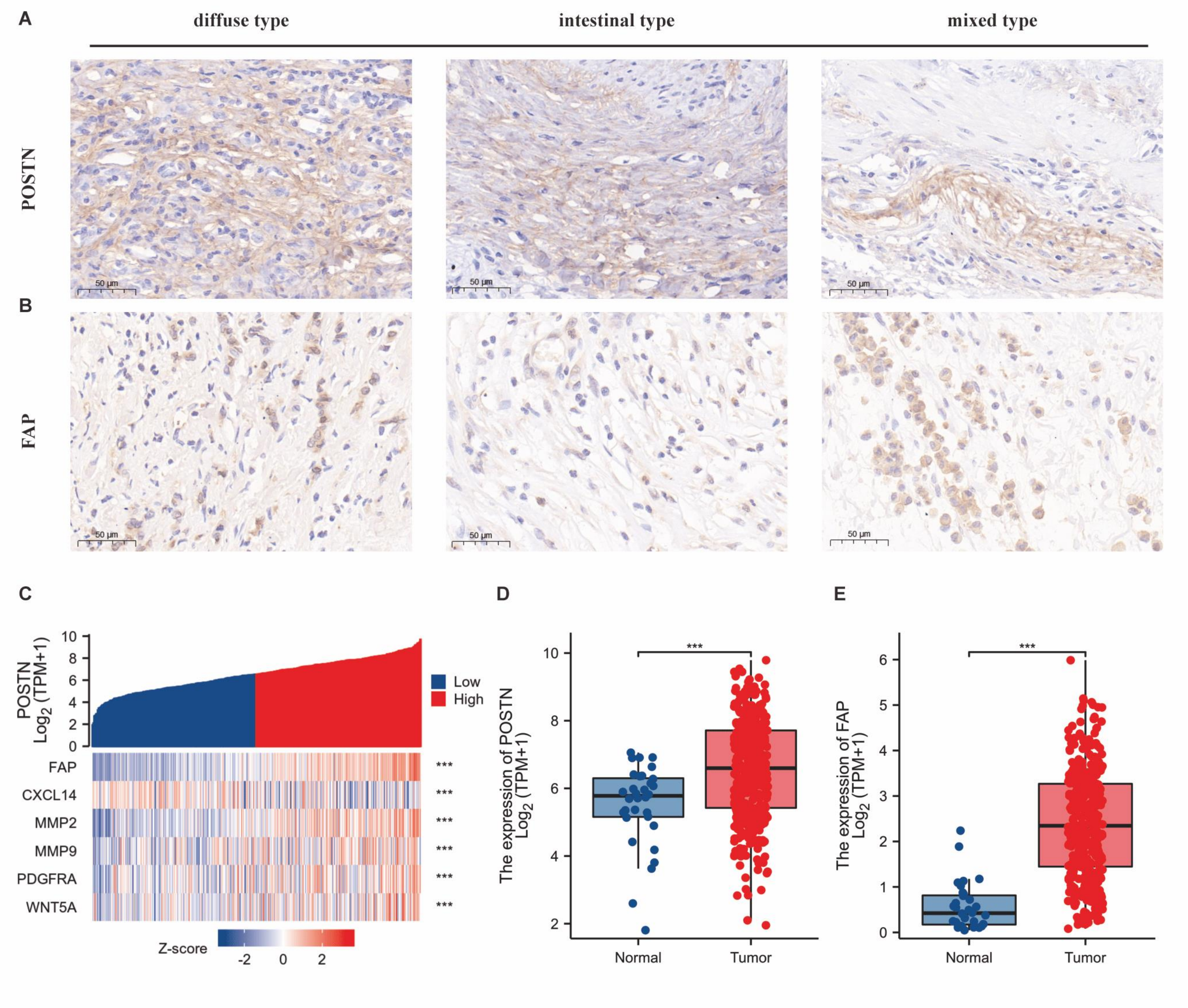

.

Supplementary Figure 1. POSTN was associated with the characteristic gene expression of CAFs. (A) The location of POSTN (A) and FAP (B) in the stromal area of diffuse-type, intestinal-type, and mixed-type gastric cancer patients by IHC (n = 22). Scale bar: 50 μm. (C) The correlation of expression levels between POSTN, FAP and other CAF characteristic genes in TCGA-STAD (***, *p*-value< 0.001)(Spearman's rank) (D-E) POSTN (D) and FAP (E) were highly expressed in GC compared with normal tissue in TCGA-STAD. Red bars represent the expression levels in tumour samples, and blue bars represent the expression levels in normal samples. (***, *p*-value< 0.001)(Wilcoxon rank sum test)

© 2023. You T et al. Published online at http://www.aginganddisease.org/EN/10.14336/AD.2023.0503

SUPPLEMENTARY DATA

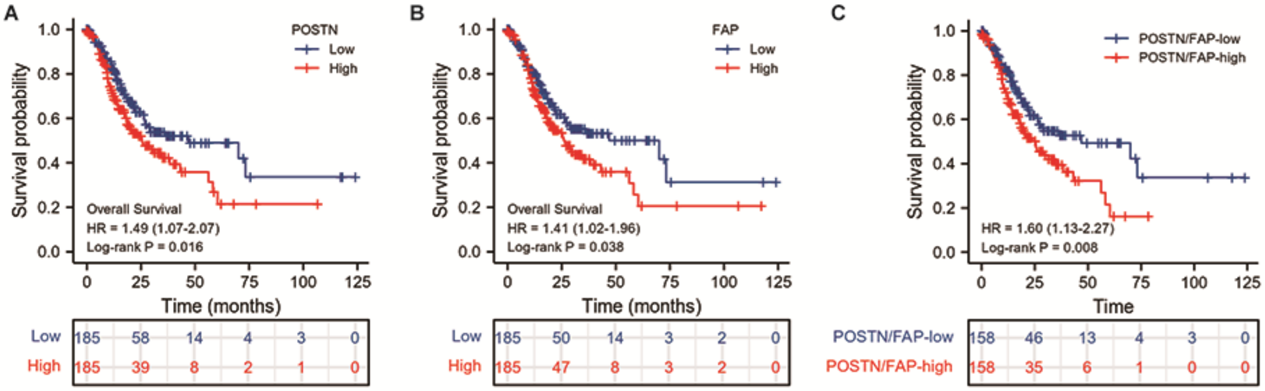

Supplementary Figure 2. POSTN⁺FAP⁺eCAFs correlated with poor prognosis in TCGA-STAD. (A-C) Kaplan–Meier survival curves revealed that high expression of POSTN (A) and FAP (B) and high expression of both POSTN and FAP (C) were correlated with short OS in GC. The top 50% expression level was defined as the high expression group, while the bottom 50% expression level was defined as the low expression group (Log-rank test).

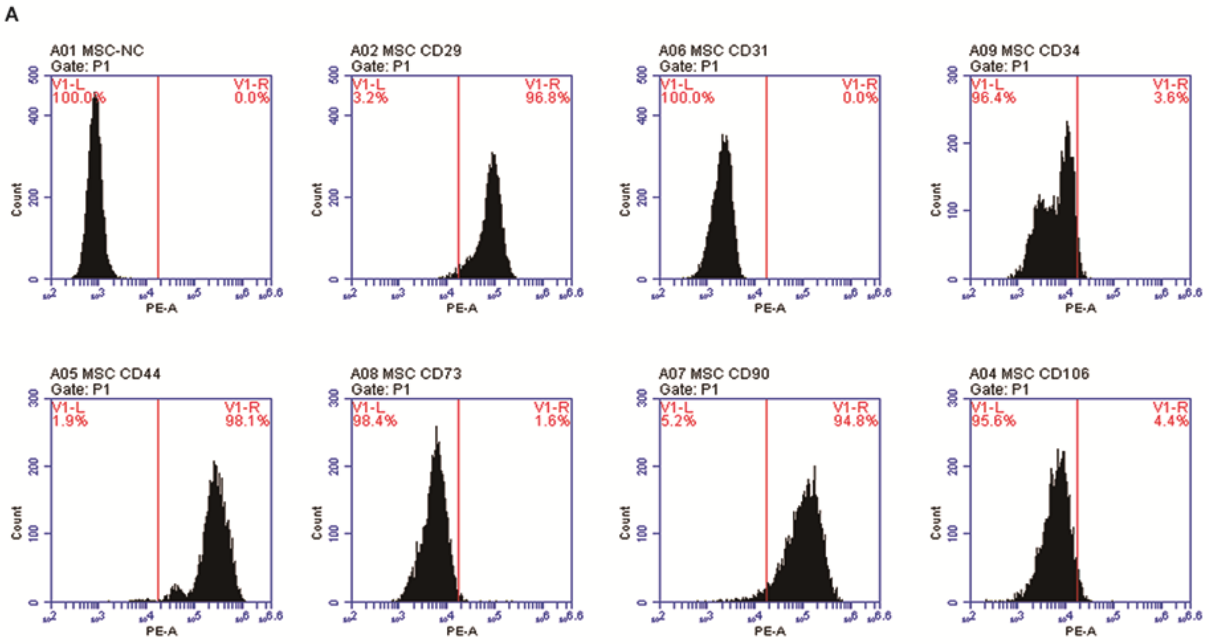

Supplementary Figure 3. The phenotypes of MSCs were identified by flow cytometry analysis.

© 2023. You T et al. Published online at http://www.aginganddisease.org/EN/10.14336/AD.2023.0503

SUPPLEMENTARY DATA

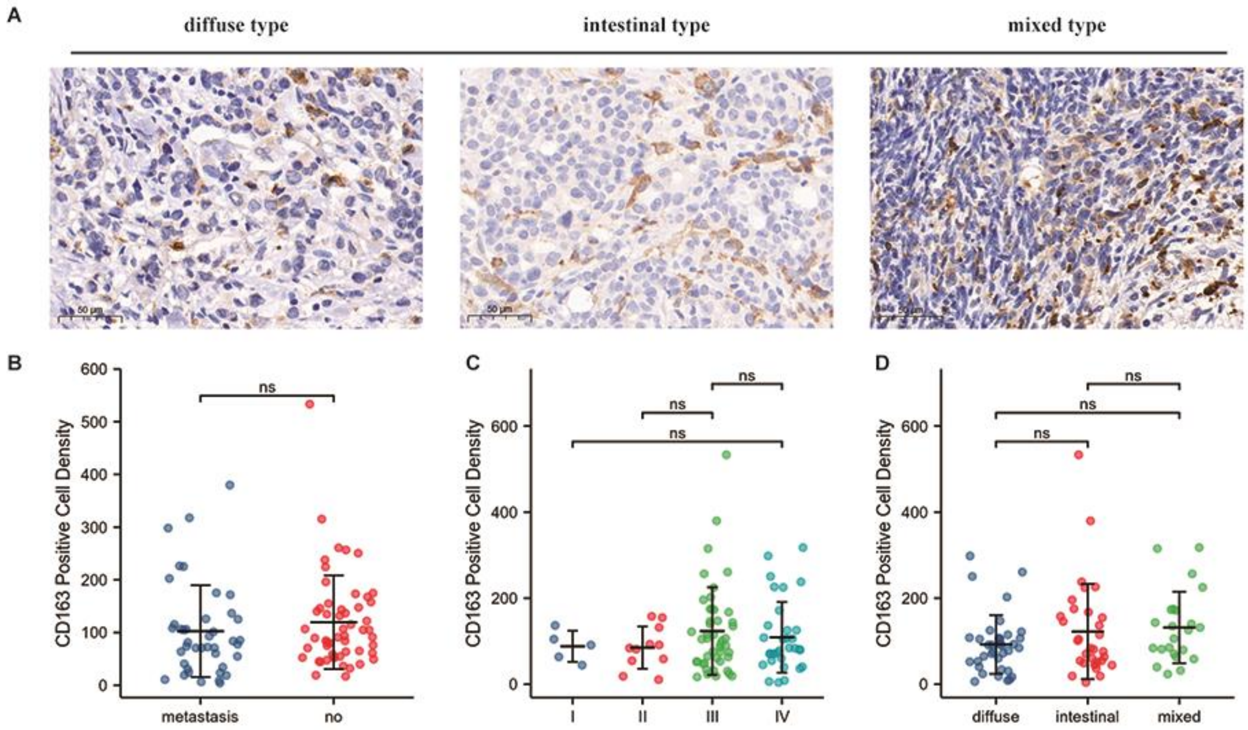

Supplementary Figure 4. The relationship of CD163 with clinicopathological features in GC patients. (A) The location of CD163 in diffuse-type, intestinal-type, and mixed-type gastric cancer patients by IHC (n = 22). Scale bar: 50 μm. (B-D) Scatter dot plots of CD163-positive cell density for patients with variable clinical features: with or without metastasis (B), stage (C), and Lauren type (D). Error bars are the mean and one standard error of the mean. (ns, no significant difference)(Kruskal-Wallis test)

SUPPLEMENTARY DATA

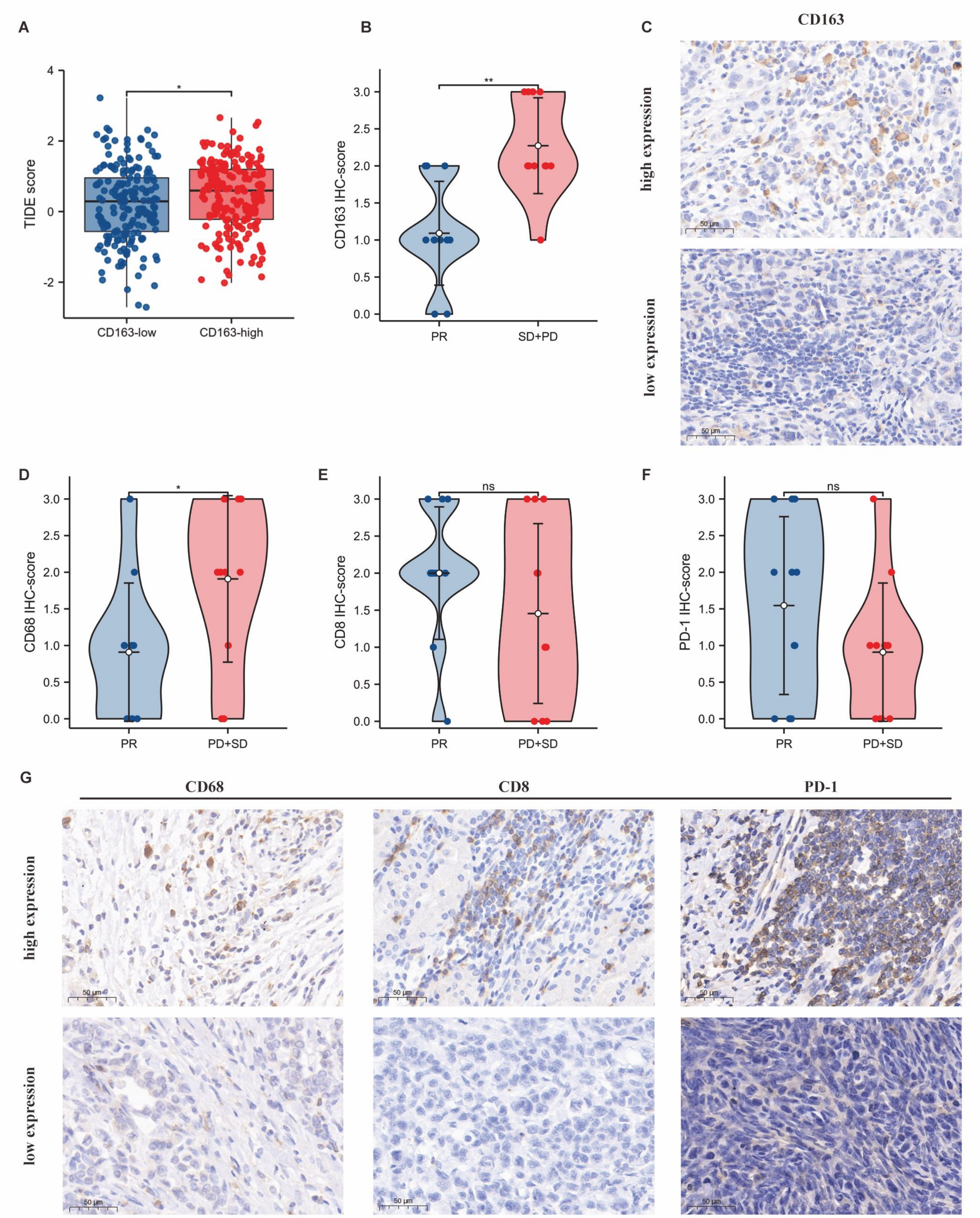

Supplementary Figure 5. High expression of CD163 conferred resistance to immune checkpoint blockade in GC. (A) The expression of CD163 was correlated with TIDE scores (*, *p*-value< 0.05)(T test). (B, C) The expression of CD163 was positively correlated with the ORR of GC patients (n = 22). B: statistical data(**, *p*-value< 0.01)(Wilcoxon rank sum test); C: IHC detected expression of CD163 in GC tumour tissue. Scale bar: 50 μm. (D-F) The relationship between the expression of CD68 (D), CD8 (E), and PD-1 (F) and the ORR of GC patients (n = 22).(**, *p*-value< 0.01; *, *p*-value< 0.05; ns, no significant difference)(Wilcoxon rank sum test). (G) IHC was used to detect the expression of CD68, CD8 and PD-1 in GC tumour tissue (n = 22). Scale bar: 50 μm.

© 2023. You T et al. Published online at http://www.aginganddisease.org/EN/10.14336/AD.2023.0503

SUPPLEMENTARY DATA

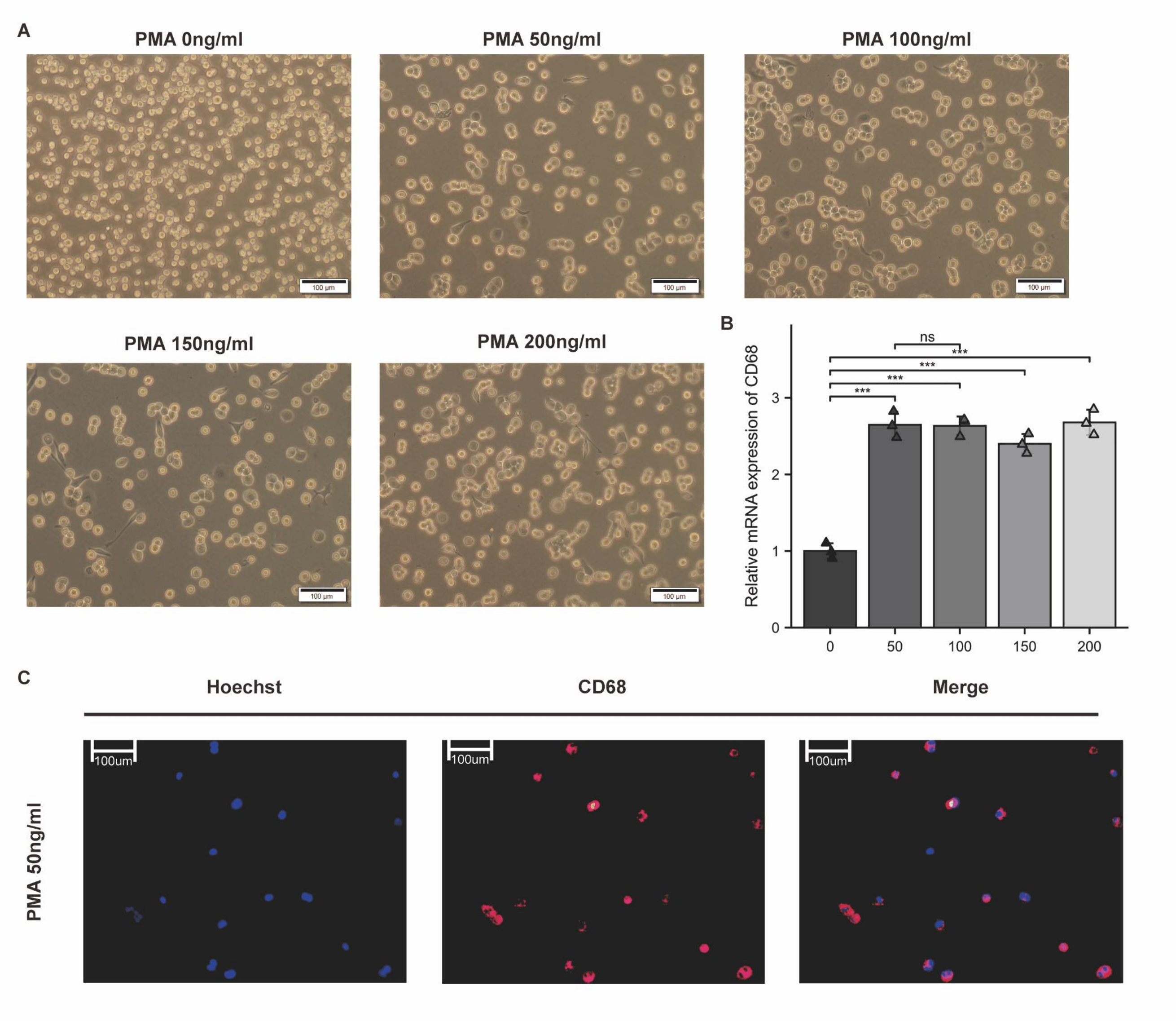

Supplementary Figure 6. Induction and identification of macrophages. (A) Microscopic micrographs showing that THP-1 cells became adherent and enlarged after stimulation with different concentrations of PMA (n = 3). (B) Quantitative PCR analysis indicated that the relative mRNA expression of CD68 was significantly upregulated after stimulation with different concentrations of PMA (***, *p*-value< 0.001; ns, no significant difference) (One-way ANOVA) (C) IF assays detected the expression of CD68 on the cell surface after stimulation with 50 ng/ml PMA (n = 3). Red: CD68; Blue: DAPI; Scale bar: 100 μm.

SUPPLEMENTARY DATA

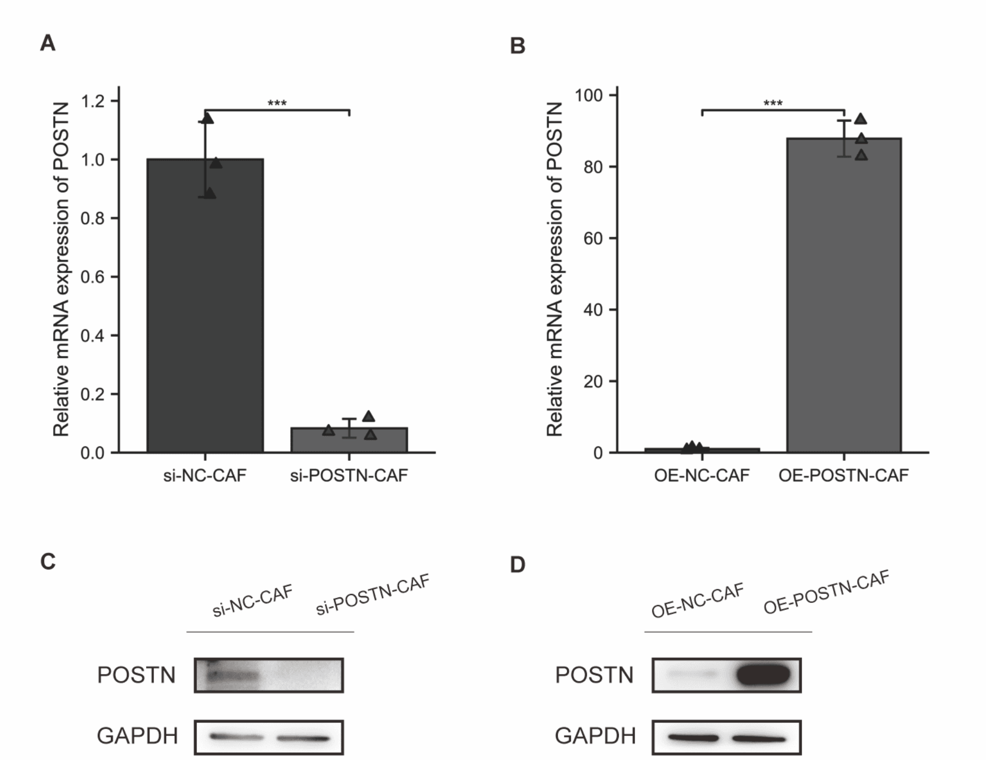

Supplementary Figure 7. Efficiency of POSTN interference and overexpression (A, C) The efficiency of POSTN interference. A: RT–PCR detected the relative mRNA expression of POSTN; C: Western blotting detected the protein expression of POSTN. si-NC-CAF: lentivirus transfection control CAFs; si-POSTN-CAF: CAFs transfected with POSTN siRNA by lentivirus. (***, *p*-value< 0.001) (One-way ANOVA). (B, D) The efficiency of POSTN overexpression. B: RT–PCR detected the relative mRNA expression of POSTN; D: Western blotting detected the protein expression of POSTN. OE-NC-CAF: lentivirus transfection control CAFs; OE-POSTN-CAF: CAFs transfected with the POSTN overexpression vector by lentivirus.

© 2023. You T et al. Published online at http://www.aginganddisease.org/EN/10.14336/AD.2023.0503

SUPPLEMENTARY DATA

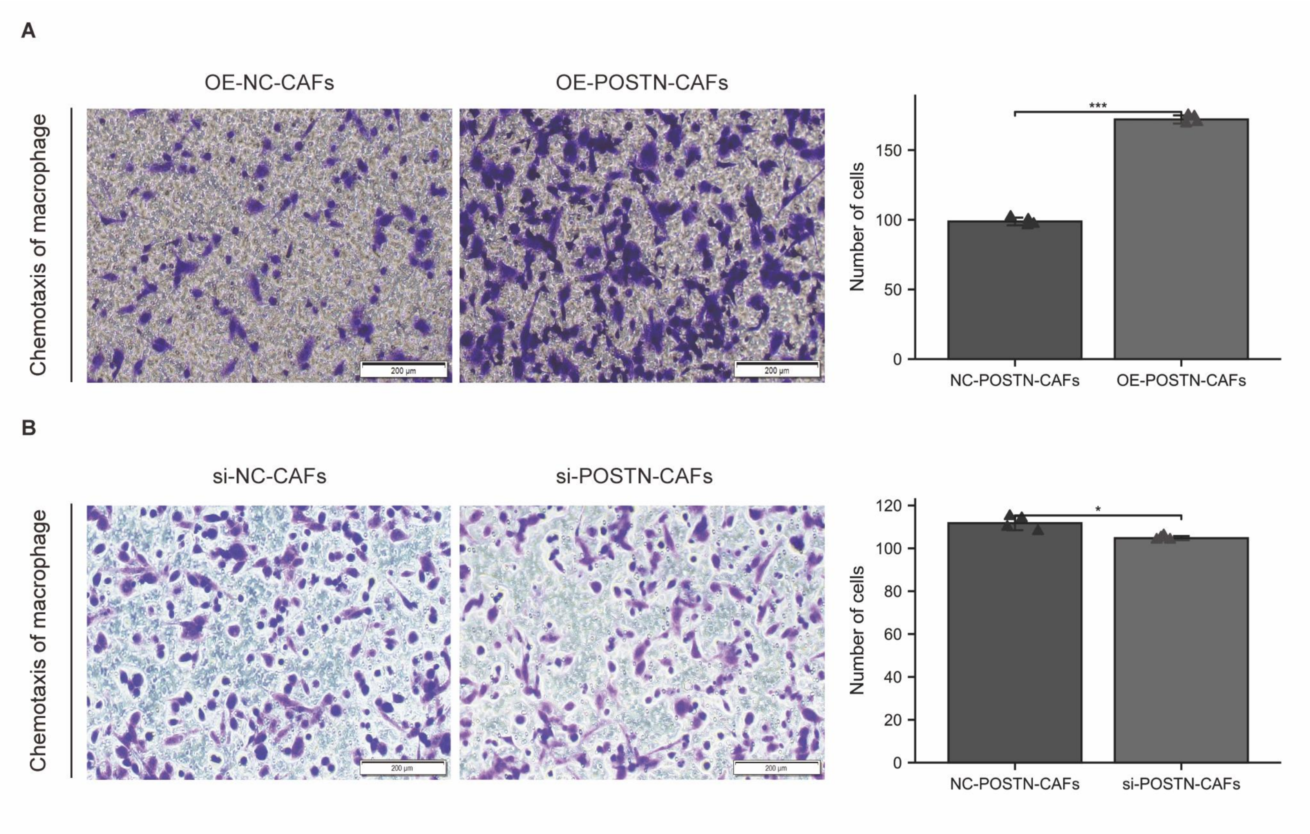

Supplementary Figure 8. Overexpression and knockdown of POSTN in CAFs influence the chemotactic ability of macrophages. A: Overpression of POSTN in CAFs enhanced the chemotactic ability of macrophages (n = 4)(***, *p*-value< 0.001)(T test). B: Knockdown of POSTN in CAFs reduced the chemotactic ability of macrophages. CAFs induced by exosomes from GC803 cell line (n = 4)(*, *p*-value<0.05)(T test). CAFs induced by exsomes from GC803 cell lines.

SUPPLEMENTARY DATA

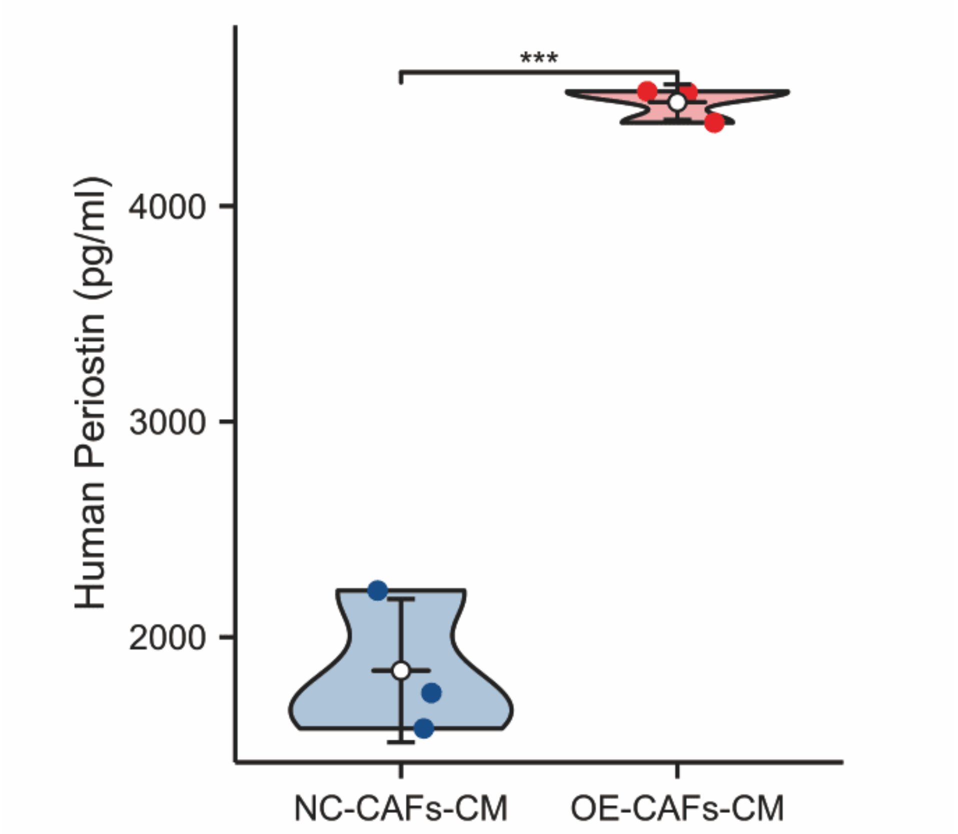

Supplementary Figure 9. ELISA was used to detect detect the POSTN level in the conditioned medium.(n=3)(***, *p*-value< 0.001)(T test).

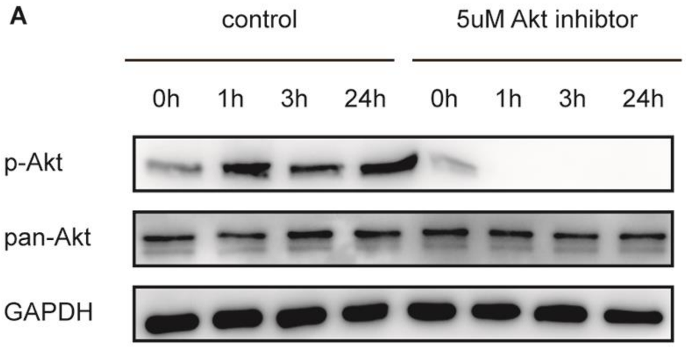

Supplementary Figure 10. Western blotting was used to detect the efficacy of an Akt phosphorylation inhibitor in macrophages.

© 2023. You T et al. Published online at http://www.aginganddisease.org/EN/10.14336/AD.2023.0503

SUPPLEMENTARY DATA

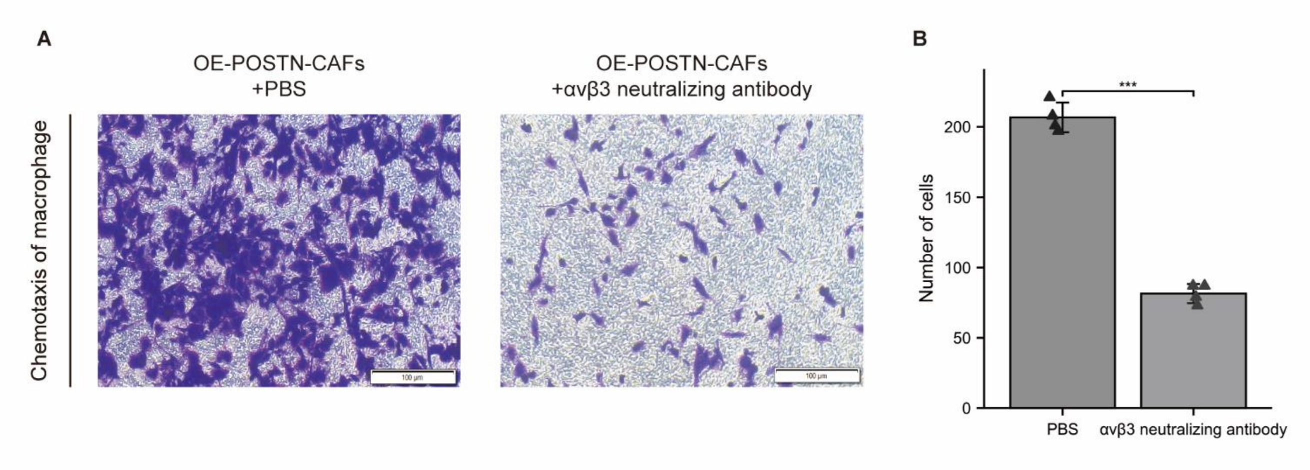

Supplementary Figure 11. Effect of αvβ5 neutralizing antibody on chemotactic macrophages of CAFs. A: Transwell assay of chemotactic macrophages (n = 4); B: statistical data (***, *p*-value< 0.001)(T test).

© 2023. You T et al. Published online at http://www.aginganddisease.org/EN/10.14336/AD.2023.0503

SUPPLEMENTARY DATA

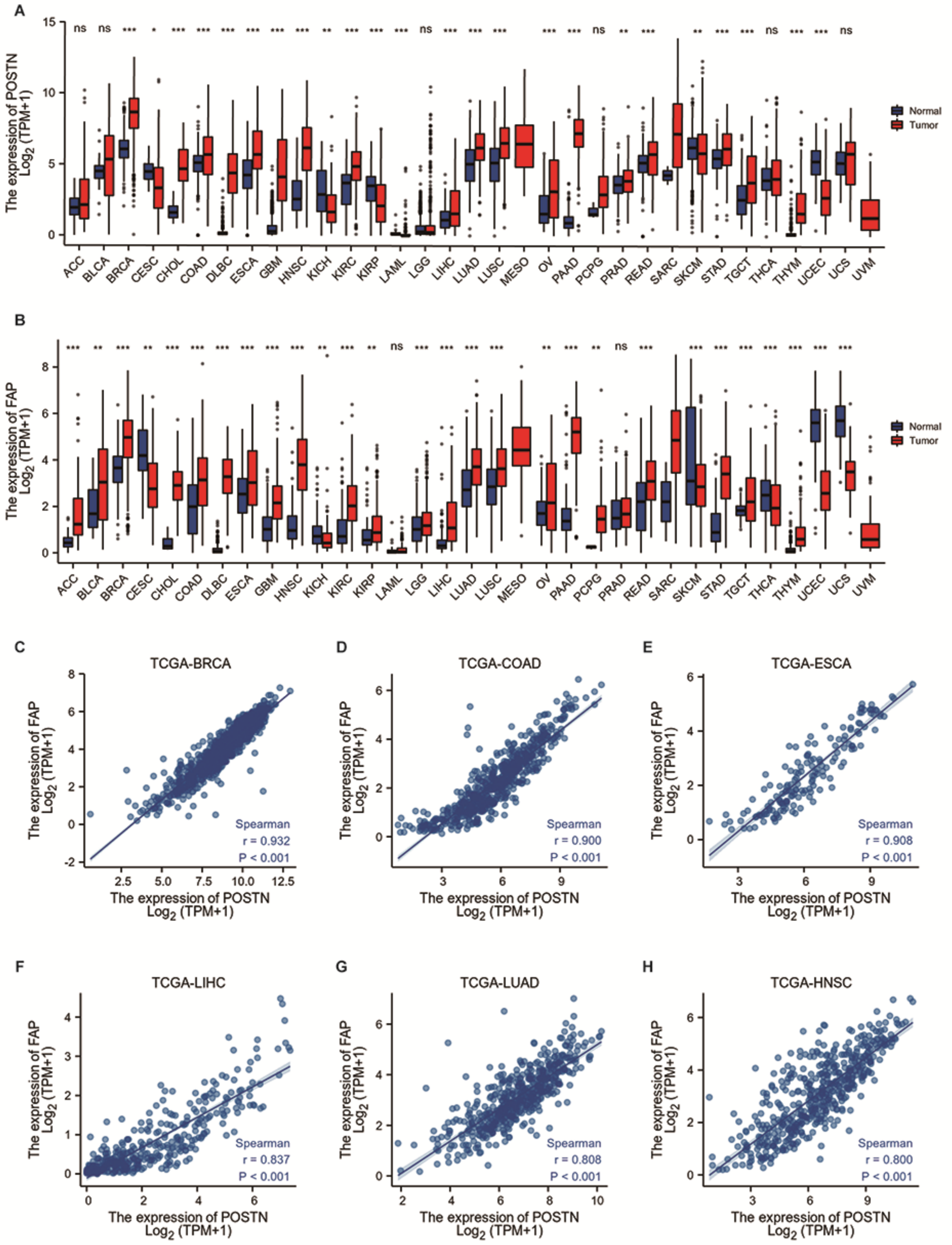

Supplementary Figure 12. Relationship between POSTN and FAP across cancers. (A)Compared to normal tissues, POSTN (A) and FAP (B) were significantly upregulated in multiple solid tumour tissues. (***, *p*-value<0.001; **, *p*-value<0.01; *, *p*-value< 0.05; ns, no significant difference)(Wilcoxon rank sum test). (C-H) POSTN expression was correlated with FAP expression in TCGA-BRCA (C), TCGA-COAD (D), TCGA-ESCA (E), TCGA-LIHC (F), TCGA-LUAD (G), and TCGA-HNSC (H). (*p*-value<0.001)(Spearman's rank)

© 2023. You T et al. Published online at http://www.aginganddisease.org/EN/10.14336/AD.2023.0503

SUPPLEMENTARY DATA

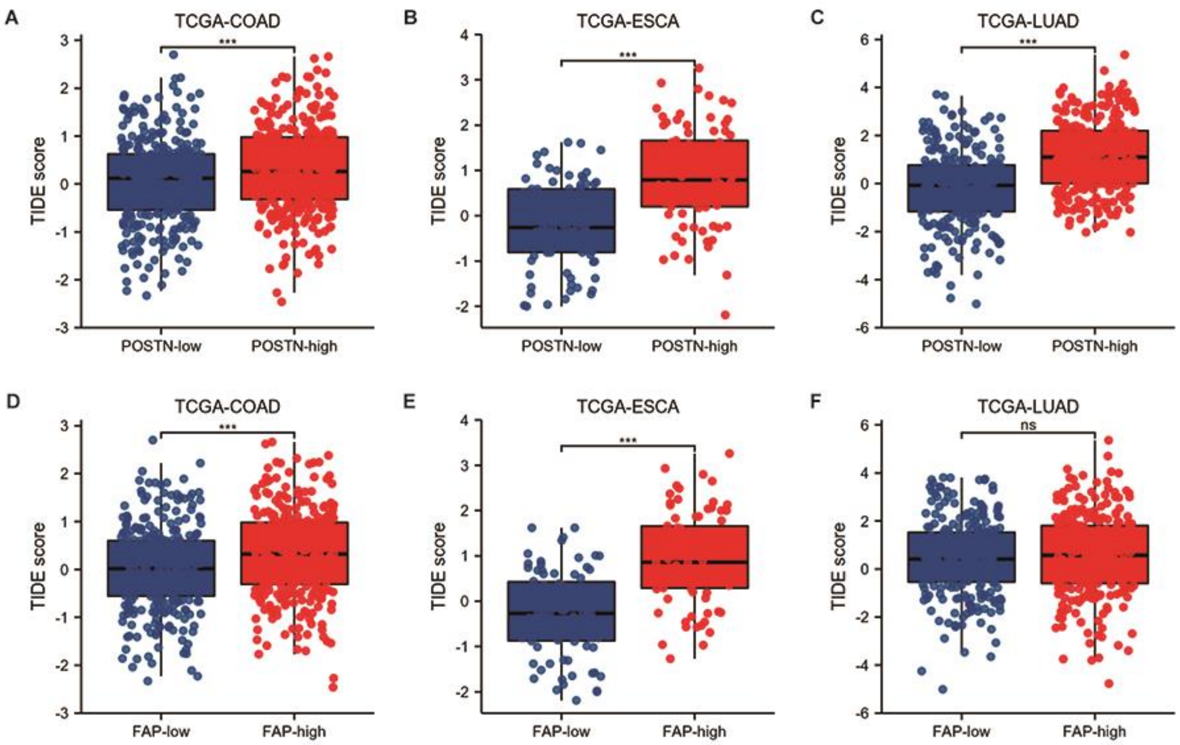

Supplementary Figure 13. POSTN^{+}FAP^{+} eCAFs correlated with ICB resistance across cancers. (A-F) TIDE scores were significantly higher in the POSTN-high (A-C) and FAP-high (D-F) groups in colon adenocarcinoma (TCGA-COAD), esophageal carcinoma (TCGA-ESCA), and lung adenocarcinoma (TCGA-LUAD). High TIDE scores indicated poor response to ICB. Red bars represent higher expression levels, and blue bars represent lower expression levels. (***, *p*-value<0.001; ns, no significant difference)(T test).

© 2023. You T et al. Published online at http://www.aginganddisease.org/EN/10.14336/AD.2023.0503

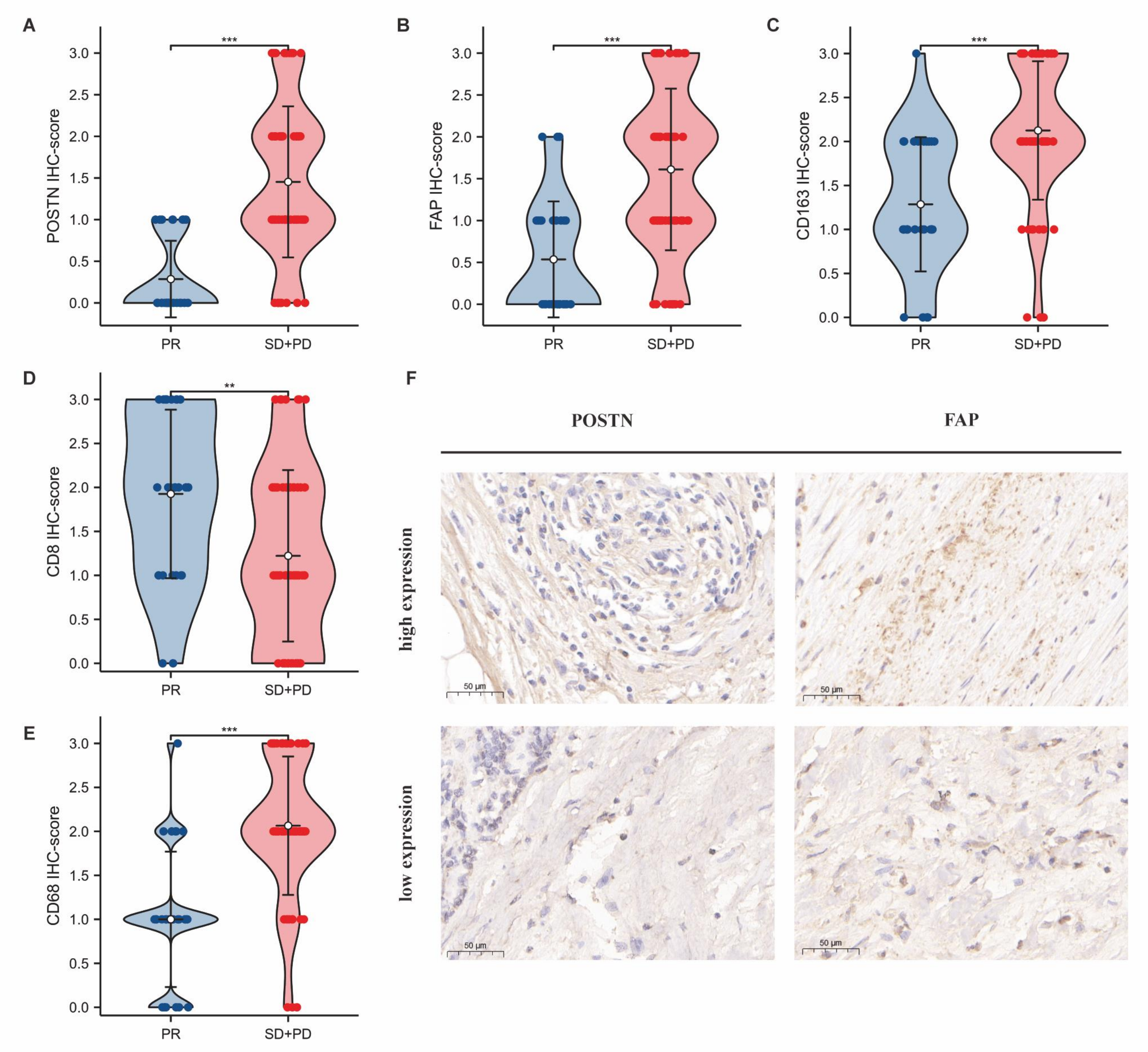

Supplementary Figure 14. POSTN⁺FAP⁺ eCAFs and TAM correlated with ICB resistance across cancers. (A-E) High expression of POSTN (A), FAP (B), CD163 (C) and CD68 (E) in GC was related to poor response to immunotherapy. PR: partial response; SD: stable disease; PD: progressive disease. Red bars represent the PD and SD groups, and blue bars represent the PR group. (***, *p*-value<0.001; **, *p*-value<0.01)(Wilcoxon rank sum test). (F) IHC staining of POSTN and FAP in GC tissue specimens (×40) (n = 92). Scale bar: 50 μm.

SUPPLEMENTARY DATA

Supplementary Table1. The baseline characteristic of 92 pan cancer patients who received chemotherapy combined with immunotherapy.

Characteristic		n (%)
n		92
age, median (IQR)		58 (51, 65)
sex	F	30 (32.6%)
	M	62 (67.4%)
primary site	lung	28 (30.4%)
	stomach	23 (25%)
	other	15 (16.3%)
	esophagus	13 (14.1%)
	liver	13 (14.1%)
stage	IV	48 (52.2%)
	III	21 (22.8%)
	II	6 (6.5%)
	I	4 (4.3%)
	NA	13 (14.1%)
T stage	Tx	31 (33.7%)
	T4	25 (27.1%)
	T3	19 (20.7%)
	T2	9 (9.8%)
	T1	8 (8.7%)
N stage	N3	10 (10.9%)
	N2	21 (22.8%)
	N1	8 (8.7%)
	N0	12 (13.0%)
	Nx	41 (44.6%)
M stage	M1	43 (46.7%)
	M0	40 (43.5%)
	Mx	9 (9.8%)
ICB response	CR	1 (1.1%)
	PD	13 (14.1%)
	PR	27 (29.3%)
	SD	51 (55.4%)

© 2023. You T et al. Published online at http://www.aginganddisease.org/EN/10.14336/AD.2023.0503

SUPPLEMENTARY DATA

Supplementary Table 2. The baseline characteristic of 22 GC patients who received chemotherapy combined with immunotherapy.

Characteristic		n (%)
n		22
age, median (IQR)		58 (48.5, 64.75)
sex	F	7 (31.8%)
	M	15 (68.2%)
stage	IV	18 (81.8%)
	III	4 (18.2%)
T stage	Tx	9 (40.9%)
	T4	9 (40.9%)
	T3	4 (18.2%)
N stage	Nx	14 (63.6%)
	N3	4 (18.2%)
	N2	3 (13.6%)
	N1	1 (4.5%)
M stage	M1	17 (77.3%)
	M0	5 (22.7%)
ICB response	PR	11 (50.0%)
	SD	8 (36.4%)
	PD	3 (13.6%)

Supplementary Table 3. The baseline characteristic of 95 patients with resectable GC.

Characteristic	High expression of POSTN	Low expression of POSTN	p-value
n	25	70	
Gender, n (%)			0.889
F	10 (10.5%)	25 (26.3%)	
M	15 (15.8%)	45 (47.4%)	
Age, meidan (IQR)	59 (47, 62)	60 (52.25, 65)	0.304
T stage, n (%)			0.474
T1	2 (2.1%)	10 (10.5%)	
T2	3 (3.2%)	10 (10.5%)	
T3	8 (8.4%)	29 (30.5%)	
T4	12 (12.6%)	21 (22.1%)	
N stage, n (%)			0.329
N0	7 (7.4%)	19 (20%)	
N1	2 (2.1%)	17 (17.9%)	
N2	6 (6.3%)	14 (14.7%)	
N3	10 (10.5%)	20 (21.1%)	
M stage, n (%)			1.000
0	14 (14.7%)	40 (42.1%)	
1	11 (11.6%)	30 (31.6%)	
Stage, n (%)			0.335
I	1 (1.1%)	4 (4.2%)	
II	1 (1.1%)	10 (10.5%)	
III	11 (11.6%)	35 (36.8%)	
IV	12 (12.6%)	21 (22.1%)	
Lauren type, n (%)			0.188
diffuse type	12 (13%)	25 (27.2%)	
iintestinal type	5 (5.4%)	27 (29.3%)	
mixed type	8 (8.7%)	15 (16.3%)	
Her-2, n (%)			0.019
0/1+	19 (21.1%)	56 (62.2%)	
2+	2 (2.2%)	10 (11.1%)	
3+	3 (3.3%)	0 (0%)	
neoadjuvant, n (%)			0.516
no	11 (11.6%)	38 (40%)	
yes	14 (14.7%)	32 (33.7%)	

© 2023. You T et al. Published online at http://www.aginganddisease.org/EN/10.14336/AD.2023.0503

SUPPLEMENTARY DATA

Supplementary Table 4. The sequences of all primers.

Gene name		Sequence
GAPDH	forward primer	TGCCATCAATGACCCCTTC
	reverse prime	CATCGCCCCACTTGATTTTG
POSTN	forward primer	TGCCCAGCAGTTTTGCCCAT
	reverse prime	CGTTGCTCTCCAAACCTCTA
	forward primer	TGCCCAGCAGTTTTGCCCAT
	reverse prime	CGTTGCTCTCCAAACCTCTA
FAP	forward primer	ATGAGCTTCCTCGTCCAATTCA
	reverse prime	AGACCACCAGAGAGCATATTTTG
CD68	forward primer	CTTCTCTCATTCCCCTATGGACA
	reverse prime	GAAGGACACATTGTACTCCACC

© 2023. You T et al. Published online at http://www.aginganddisease.org/EN/10.14336/AD.2023.0503
